# Relevant Probiotic and Functional Properties of Lactic Acid Bacteria Isolated from Aquaculture Environments on the Ivory Coast for Potential Aquaponic Applications

**DOI:** 10.3390/microorganisms14091906

**Published:** 2026-08-28

**Authors:** Wahauwouélé Hermann Coulibaly, Tano Marie-Ange Sakia Mian, Yabo Majoie Géroxie Tohoyessou, Muiz O. Akinyemi, Bassey Ebenso, Ange Olivier Parfait Yao, Cécile Meex, Paul-Alexandru Popescu, Thierry Fievez, Phillipe Maesen, Hary Razafindralambo

**Affiliations:** 1 Biotechnology and Food Microbiology Laboratory Food Science, Technology Formation and Research Unit, Nangui Abrogoua University, Abidjan 02 BP 801, Côte d’Ivoire; wahauwouele@yahoo.fr; 2 Agro-Valorization Laboratory, Agroforestery Trainning and Research Unit, Jean Lorougnon Guédé University, Daloa BP 150, Côte d’Ivoire; 3 Biology and Molecular Typage in Microbiology Laboratory, Biochemistry and Cell Biology Department, Faculty of Sciences and Techniques, University of Abomey-Calavi, Cotonou 05 BP 1604, Benin; 4 Leeds Institute of Health Sciences, University of Leeds, Leeds LS2 9LN, UK; m.o.akinyemi@leeds.ac.uk (M.O.A.); b.e.ebenso@leeds.ac.uk (B.E.); 5 Unit for Environmental Sciences and Management, North-West University, Potchefstroom 2531, South Africa; 6 Microbial Processes and Interactions, TERRA Teaching and Research Centre, UMRt 1158 BioEcoAgro, Gembloux Agro-Bio Tech, University of Liege, 5030 Gembloux, Belgium; 7 Clinical Microbiology Laboratory, University Hospital of Liege, University of Liege, 4000 Liege, Belgium; c.meex@chuliege.be; 8 Faculty of Biotechnology, University of Agronomic Sciences and Veterinary Medicine of Bucharest, 59 Marasti Blvd, District 1, 011464 Bucharest, Romania; 9 BEAGx, Gembloux Agro-Bio Tech, University of Liege, 2 Passage Des Déportés, 5030 Gembloux, Belgium; 10 ProBioLab, 5004 Namur, Belgium; 11 Faculty of Agriculture and Natural Resources, An Giang University, Long Xuyen 90100, An Giang, Vietnam; 12 Ingénierie et Technologie Emergentes, Université de Vakinankaratra, Campus Vatofotsy, Antsirabe 110 BP 108, Madagascar

**Keywords:** beneficial microbes, metabolic profiling, plant growth-promoting rhizobacteria (PGPR), nitrification, *Enterococcus faecalis*, *Oreochromis niloticus*

## Abstract

Aquaponics combines aquaculture and hydroponics, offering an integrated and sustainable food production system. This study investigated the probiotic properties, plant growth-promoting (PGP) activity, and nitrifying capacity of twelve lactic acid bacteria (LAB) strains isolated from an aquaculture farm environment on the Ivory Coast for their potential application in aquaponic systems. All isolates demonstrated antagonistic activity against key pathogenic indicator strains, except *Vibrio cholerae*, and displayed varying levels of surface hydrophobicity (5.50 ± 0.70% to 22.83 ± 1.17%) and auto-aggregation (32.50 ± 0.08% to 52.89 ± 0.39%) after 24 h. Antioxidant activity was significantly higher in cell-free supernatants (~71–79%) than in intact cells (~30–33%). Bile salt tolerance (0.3%, 4 h) ranged from 2.13 ± 0.76% to 40.87 ± 2.12%, and survival under pH 1.5 with pepsin for 3 h varied from 4.84 ± 0.26% to 53.98 ± 13.28%. All isolates produced lactic, acetic, citric, malic, and propionic acid and exhibited amylase and cellulase activity; none showed hemolytic activity. Only LAB 11 produced indole-3-acetic acid (17.72 ± 0.06 μg/mL), siderophores, and phosphate-solubilization activity for PGP traits, and this group significantly enhanced maize seed germination (86.66 ± 5.77%) and radicle length (7.00 ± 0.52 mm) compared to the control (63.33 ± 32.14% and 4.94 ± 1.52 mm), respectively. LAB 1 and LAB 10 demonstrated the highest ammonia-oxidizing capacity in vitro and in trout pond water. LAB 1, LAB 10, and LAB 11 were confirmed by whole genome sequencing analysis to be *Enterococcus faecalis* strains with a favorable-safety genomic profile and probiotic characteristics. These three strains therefore represent promising candidates for consortium-based applications in aquaponics systems.

## 1. Introduction

The global population, currently estimated at approximately 8 billion, is projected to reach 9.8 billion by 2050 [1]. This demographic pressure, combined with the intensification of agricultural and aquacultural practices, poses significant challenges for food security and environmental sustainability. Aquaponics, an integrated production system that couples recirculating aquaculture with hydroponics, has emerged as a promising solution that can reduce water consumption, eliminate the need for synthetic fertilizers, and simultaneously produce fish and vegetables in a single closed loop [2].

In many developing nations, including the Ivory Coast, aquaculture farms depend heavily on low-cost agricultural by-products such as fish feed, while the widespread use of antibiotics to control bacterial diseases increasingly drives antimicrobial resistance (AMR), threatening both fish health and consumer safety [3,4,5]. Global aquaculture production reached 82 million tons in 2018, representing 46% of total fish production, and continues to expand, placing intensifying pressure on sustainable disease management strategies [6]. The use of probiotic bacteria represents an ecologically sound and economically viable alternative to antibiotics in aquaculture [7,8,9]. Comprehensive recent reviews confirm that probiotics enhance disease resistance, immunity, digestive performance, and water quality in diverse aquatic species, including tilapia, shrimp, and salmonids, while substantially reducing reliance on chemotherapeutics [6,10,11,12].

Probiotics are defined as live microorganisms that, when administered in adequate quantities, confer a health benefit on the host [13]. Among these, lactic acid bacteria (LAB), including *Lactobacillus* spp., *Bifidobacterium* spp., *Pediococcus* spp., and *Enterococcus* spp., have been widely studied for their beneficial effects in aquaculture, including improved feed conversion, enhanced digestive enzyme activity, stimulation of the immune system, better water quality, and reduced disease incidence [10,11,12,14,15]. A growing body of evidence suggests that probiotic efficacy is context-dependent, and that locally isolated strains colonize the fish gut more effectively than non-native strains [16,17].

Beyond aquaculture, LAB are increasingly recognized as plant growth-promoting rhizobacteria (PGPR) with significant potential for sustainable agriculture [18,19,20]. PGP mechanisms include biofertilization (phosphate and potassium solubilization, biological nitrogen fixation), phytostimulation through phytohormone production (notably indole-3-acetic acid, IAA, and gibberellic acid), bioprotection via hydrolytic enzyme and siderophore secretion, and bioremediation of heavy metals [21,22,23]. In the context of aquaponics, a bacterial strain that combines probiotic, PGP, and nitrifying properties could simultaneously benefit fish health, promote plant growth through nutrient provision, and contribute to biological water treatment by converting toxic ammonia into plant-available nitrate, thereby offering a sustainable, multifunctional solution that reduces the need for external chemical inputs and enhances overall system productivity. *Enterococcus* spp. have recently gained attention as dual-function organisms. Pruthviraj et al. [24] characterized *Enterococcus faecium* MYSBC14 in terms of its combined antibacterial, probiotic, and PGP attributes, reporting IAA production of 11.56 µg/mL alongside gibberellic acid production (10.18 µg/mL), phosphate and potassium solubilization, and siderophore production. In a parallel line of research, Batool et al. [25] demonstrated that auxin-producing *Enterococcus* sp. SR9 could be leveraged for the biogenic synthesis of silver nanobiofertilizers (SR9AgNPs), achieving a 98% germination rate and a vigor index of 1928 in wheat seedlings, substantially higher than untreated controls, thereby highlighting the broader agricultural potential of this genus. LAB-based biofertilizers have further been shown to improve soil microbiome composition, chlorophyll content, and yield in crops like tomato [26], underscoring their promise as multifunctional agents in integrated bio-agricultural systems.

Despite the growing interest in LAB for aquaponic application, studies that comprehensively characterize strains across all three functional dimensions, probiotic, PGP, and nitrifying, remain scarce, particularly for strains of African aquaculture origin. Di Benedetto et al. [27] highlighted the need for multi-trait PGP selection frameworks that explicitly include nitrification capacity alongside phosphate solubilization, siderophore production, and IAA biosynthesis, yet such frameworks have rarely been applied to LAB from aquaculture environments. The present study aimed to screen twelve LAB isolates from a tilapia (*Oreochromis niloticus*) farm on the Ivory Coast for (i) key probiotic traits, including antibacterial activity, GI tolerance, antioxidant capacity, cell-surface properties, enzyme production, and safety characteristics; (ii) PGP traits, including IAA and siderophore production, phosphate and potassium solubilization, and ammonia production; and (iii) nitrifying capacity (ammonia oxidation to nitrite and nitrate), both in vitro and in trout pond water. Three strains fulfilling criteria across multiple categories (LAB1, LAB10, and LAB11) were identified by 16S rRNA sequencing, and were confirmed and phylogenetically characterized via whole genome sequencing analysis as *Enterococcus faecalis* with a favorable safety genomic profile and genomic characteristics consistent with recognized probiotic organisms [28].

## 2. Materials and Methods

### 2.1. Bacterial Strains and Culture Conditions

Twelve LAB isolates were obtained from the extraction of pond water from the aquaculture farm at the University Nangui Abrogoua, Ivory Coast, and plating on de Man, Rogosa and Sharpe (MRS) agar (Biokar Diagnostics, Allonne, France), as described previously [29]. Briefly, pond water samples were serially diluted in sterile saline solution, spread onto MRS agar plates, and incubated at 37 °C for 48 h. Colonies displaying typical LAB morphology were purified by repeated streaking on MRS agar and selected for further analysis. Isolates were stored at −20 °C in 20% (*v*/*v*) glycerol until use. Working cultures were prepared through two successive transfers in MRS broth (37 °C, 48 h, 150 rpm) prior to each assay.

### 2.2. Probiotic Characterization

#### 2.2.1. Antibacterial Activity

Antibacterial activity was assessed via a well-diffusion assay [30]. After 48 h of culture in MRS broth (37 °C, 150 rpm), cultures were centrifuged (10,000× *g*, 4 °C, 5 min) and filtered through 0.22 μm Millipore membranes to yield cell-free supernatants (CFSs). Nutrient agar plates were inoculated with 100 μL of each indicator strain (OD_600_ = 0.2; ~10^8^ CFU/mL): *Pseudomonas aeruginosa* ATCC 27853, *Escherichia coli* ATCC 25922, *Vibrio cholerae* ATCC 7253, *Aeromonas hydrophila* subsp. *hydrophila* LM 92844, and *Staphylococcus aureus* ATCC 29213. Wells of 6 mm diameter were punched under aseptic conditions, loaded with 100 μL CFS, and incubated at 37 °C for 24 h. Inhibition zones ≥1 mm were recorded as positive. All assays were performed in triplicate.

#### 2.2.2. Gastrointestinal Tolerance

Bile salt tolerance was assessed according to Diguţă et al. [31] with minor modifications. Each LAB suspension (OD_600_ = 0.3; ~10^8^ CFU/mL) was inoculated into MRS broth supplemented with 0.3% (*w*/*v*) bile salts (Sigma-Aldrich Chemie GmbH, Steinheim, Germany) and incubated at 37 °C for 4 h. Viable counts were determined by plate count at 0 h and 4 h; growth rate was calculated as

(1) $$\left[\frac{logCFU\;final}{log\;CFU\;initial}\right]\times 100$$

Resistance to pepsin under simulated gastric conditions was determined by resuspending cell pellets (4500× *g*, 10 min) in PBS containing 0.3% (*w*/*v*) pepsin, adjusting the pH to 1.5 with 1 N HCl, and incubating at 37 °C for 3 h. Survival rate was calculated as in the case of bile salt tolerance. All assays were performed in triplicate.

#### 2.2.3. Antioxidant Activity (DPPH Assay)

DPPH radical-scavenging activity was measured according to Brand-Williams et al. [32]. Following overnight incubation in MRS broth (37 °C), cell suspensions were centrifuged (4500× *g*, 30 min, 4 °C). Supernatant (CFS) and intact cell pellets (resuspended in PBS, OD_600_ = 0.3 ± 0.05) were mixed 1:2 with DPPH reagent (100 μM in methanol) and incubated in the dark at room temperature for 30 min, and absorbance was measured at 517 nm.

(2) $$Scavenging\;activity\;(\%)=\left[\frac{{{OD}^{DPPH}-OD}^{Sample}}{{OD}^{DPPH}}\right]\times 100$$

Deionized water served as a negative control. All measurements were performed in triplicate.

#### 2.2.4. Cell Surface Properties

Intestinal cell surface hydrophobicity was determined by the microbial adhesion to hydrocarbons (MATH) method using chloroform and hexane as apolar solvents, as originally described by Rosenberg et al. [33], with minor modifications. Cell suspensions (OD_600_ = 0.3 ± 0.05) were vortexed with a solvent (2.4:0.4 ratio) for 2 min and allowed to phase-separate for 30 min, and the aqueous-phase absorbance was measured at 600 nm (H_1_).

(3) $$Hydrophobicity\;(\%)=\left[\frac{{(H}_{0}-H_{1})}{H_{0}}\right]\times 100$$

Auto-aggregation (ability to form biofilm is the gastrointestinal tract) was assessed after 2 h and 24 h of incubation at 37 °C following Polak-Berecka et al. [34]; the coefficient was calculated as

(4) $$Auto-aggregation\;(\%)=\left[\frac{{(ODvalue}_{initial}-{ODvalue}_{time})}{{ODvalue}_{initial}}\right]\times 100$$

#### 2.2.5. Organic Acid Profiling

LAB cultures (OD_600_ = 0.3, MRS broth, 48 h, 37 °C, 150 rpm) were centrifuged, and supernatants were filtered (0.22 μm). The production of organic acids by the LAB strains was quantified by HPLC (Agilent 1200 Series, Agilent Technologies, Santa Clara, CA, USA) equipped with a refractive index detector. Chromatographic separation was carried out on an Aminex^®^ HPX-87H column (300 × 7.8 mm, Bio-Rad, Hercules, CA, USA) using 5 mM sulfuric acid as the mobile phase at a flow rate of 0.6 mL min^−1^. A sample volume of 20 μL was injected, and analyses were completed within 35 min. Organic acids were quantified from calibration curves prepared with authentic standards and analyzed using Agilent OpenLab CDS software (v2.3). Citric, malic, acetic, propionic, and lactic acids were successfully identified and quantified.

#### 2.2.6. Enzyme Production

Amylase, cellulase, lipase, and protease activities were assessed by inoculating LAB on selective solid media: (a) MRS agar with 1% soluble starch for amylase, revealed by Lugol’s iodine [35]; (b) MRS agar with 1% carboxymethylcellulose for cellulase, revealed by 0.1% Congo red/1 M NaCl wash [36]; (c) MRS agar with Tween 80/CaCl_2_/rhodamine for lipase, revealed under UV fluorescence at 350 nm [37]; and (d) skim-milk agar for protease [38]. Plates were incubated at 37 °C for 4 days.

#### 2.2.7. Safety Characteristics

Antibiotic susceptibility was evaluated by disk diffusion on MRS agar according to CLSI guidelines [39]. Twelve antibiotics representing eight classes were tested: Minocycline (30 μg), Tetracycline (30 μg), Ampicillin (10 μg), Cefotaxime (5 μg), Penicillin (1 μg), Amoxicillin–Clavulanate (30 μg), Rifampicin (5 μg), Chloramphenicol (30 μg), Ciprofloxacin (5 μg), Gentamicin (10 μg), Erythromycin (15 μg), and Amikacin (30 μg). All antibiotics were provided by Becton Dickinson (Covington, GA, USA). Results were interpreted according to the CLSI (2016) breakpoints. Hemolytic activity was assessed on Columbia agar with 5% sheep blood after 48 h at 37 °C; a β-hemolytic *Streptococcus* sp. served as the positive control.

### 2.3. Plant Growth-Promoting (PGP) Traits

#### 2.3.1. IAA Production

Qualitative IAA production was screened on LB agar without tryptophan, added using the Salkowski reagent assay (filter paper method, 5-day incubation at 37 °C) [40]. Positive strains were grown in LB broth (37 °C, 48 h, 150 rpm) and centrifuged (4500× *g*, 30 min, 4 °C), and supernatants were mixed 1:1 with the Salkowski reagent. Absorbance at 536 nm was converted to IAA concentration using a standard curve (5–100 μg/mL). All measurements were performed in triplicate.

#### 2.3.2. Siderophore Production

Qualitative screening was performed on Chrome Azurol Sulfonate (CAS) agar [41]; positive strains were quantified in succinate liquid medium using the CAS-Fe shuttle assay [42].

(5) $$Siderophore\;units\;(SU\%)=\left[\frac{{(A}_{r}-A_{s})}{A_{r}}\right]\times 100$$

where Aᵣ is the reference absorbance (uninoculated CAS solution) and A_s_ is the sample absorbance at 630 nm.

#### 2.3.3. Phosphate and Potassium Solubilization

Phosphate solubilization was tested on NBRIP agar plates (National Botanical Research Institute’s Phosphate) as a medium, and for potassium solubilization, bromothymol blue-modified Aleksandrov media was used. The media were inoculated with 20 μL per strain (OD_600_ = 0.3), and incubated at 37 °C for 5 days, as described by Nautiyal [43] and Rajawat et al. [44]. Halo diameter (mm) was measured in triplicate.

#### 2.3.4. Ammonia Production

Ammonia production was detected using the Nessler reagent colorimetric method in peptone water (1%). A volume of 100 μL of each bacterial strain was inoculated in 10 mL of peptone water and incubated for 4 days at 37 °C with 150 rpm agitation. After incubation, 0.5 mL of Nessler reagent was added in each tube. Yellowish or brownish coloration indicated a positive result [45].

### 2.4. Nitrifying Capacity

#### 2.4.1. In Vitro Assay

In vitro ammonia oxidation was quantified using the liquid ammonia oxidation medium described by Li et al. [46]. Cultures were inoculated to an initial OD_600_ = 0.3 and incubated at 37 °C for 7 days with shaking at 150 rpm. The ammonia oxidation medium was prepared as follows: (1) dissolve 4.125 g ammonium sulfate, 0.5167 g potassium dihydrogen phosphate, 0.0251 g magnesium sulfate, and 0.0019 g calcium chloride in 1 L of water, then add 2.28 mg ferrous sulfate and 1.07 mg copper sulfate with continuous stirring; (2) prepare a phosphate buffer by mixing potassium dihydrogen phosphate, sodium dihydrogen phosphate and ultrapure water at a weight ratio 8.2:0.7:300 and stir thoroughly; (3) adjust the pH of the buffer to 7.8–8.2 by adding 10 N sodium hydroxide; (4) combine the solutions from steps (1) and (3) at a volume ratio of 4:1 and sterilize; and (5) separately sterilize a 66.67 g/L sodium bicarbonate solution, then aseptically add it to the sterilized mixture to obtain a final sodium bicarbonate of 1.8–2.2 g/L. Mix thoroughly to obtain the ammonia-oxidizing culture medium.

#### 2.4.2. In Vivo Assay

For in vivo validation, LAB 1 and LAB 10, selected based on the in vitro results, were inoculated into 250 mL volumes of trout pond water (OD_600_ = 0.3) and incubated at ambient temperature for 7 days without water exchange. Ammonia, nitrite, and nitrate were quantified using Nanocolor^®^ kits (Macherey-Nagel SAS, Strasbourg, France). Controls consisted of uninoculated peptone water. All measurements were performed in triplicate.

### 2.5. Maize Seed Germination Test

Maize (*Zea mays* L.) seeds were chosen for the germination test due to their large size, ease of handling, and rapid, uniform germination. These characteristics facilitate accurate monitoring of germination parameters and improve the reproducibility of the experimental results. The LAB strain exhibiting relevant properties regarding the PGPR tests was chosen for the germination test. The maize seed germination test was carried out according to the method described by Monjezi et al. [47]. LAB 11 strain culture was carried out in LB broth with 1% glucose for 96 h at 37 °C. Surface-sterilized maize seeds (70% ethanol, 5 min; three rinses with sterile deionized water), and 10 seeds were placed on filter paper in Petri dishes moistened with either 5 mL of the CFS from LAB 11 (diluted 1:50 in sterile water; LB broth with 1% glucose, 96 h, 37 °C) or 5 mL sterile water (control). Dishes were incubated at 20 °C (70% RH, 24 h dark). Germination rate (%), expressed as the ratio of the number of germinated seeds to the total number of seeds, and radicle length (mm) were recorded after 96 h in triplicate.

(6) $$Germination\;rate\;(\%)=\left[\frac{GS}{TS}\right]\times 100$$

GS: number of germinated seeds
TS: Total number seeds

### 2.6. Molecular Identification

Molecular identification of the selected strains (LAB 1, LAB 10, and LAB 11) was performed via 16S rRNA sequencing using universal primers 27F (5′-AGAGTTTGATCMTGGCTCAG-3′) and 1492R (5′-TACGGYTACCTTGTTACGACTT-3′). PCR was carried out in a total volume of 50 µL. PCR conditions: Initial denaturation at 95 °C for 3 min; 35 cycles of 94 °C (1 min), 55 °C (1 min), 72 °C (1 min); final extension at 72 °C for 7 min. Amplicons were purified and sequenced bidirectionally (CEMIA, Larissa, Greece) and deposited in the NCBI GenBank database under accession numbers PX518070–PX518072.

Phylogenetic analysis was conducted in R (version 4.5.3) using a custom pipeline. The whole genome of the highly similar reference 16S rRNA gene sequences of *Enterococcus*-type strains was retrieved from the NCBI GenBank database using the rentrez package (v1.2.3). Sequences were aligned using the DECIPHER package (v2.28.0), with the AlignSeqs function employing the default DNA substitution matrix. The alignment was trimmed to remove poorly aligned regions using the trimAlignment function. Evolutionary distances were calculated using the Kimura 2-parameter (K2P) model with the dist.dna function from the ape package (v5.7-1), accounting for transition/transversion bias. A neighbor-joining (NJ) phylogenetic tree was constructed using the nj function (ape package) and rooted with *Staphylococcus haemolyticus* (accession L37600.1) as the outgroup. To visualize sequence similarity relationships and clustering patterns, an N-Locus Variant (NLV) graph was generated using the igraph package (v1.5.1). The genome annotation of the isolates in this study, including functional gene calls and detailed descriptions of probiotic and virulence genes, is reported in full in a comparison study [28].

### 2.7. Statistical Analysis

All statistical analyses were performed using Graphpad Prism 10 except where stated otherwise. Data are presented as means ± standard deviation (SD) of three replicates. Prior to parametric testing, data normality was assessed using the Shapiro–Wilk test and homogeneity of variance was verified with Levene’s test. Inter-strain differences for all quantitative parameters (organic acids, DPPH radical-scavenging activity, bile salt tolerance, acid–pepsin survival, cell-surface hydrophobicity, auto-aggregation, IAA concentration, siderophore units, potassium halo diameter, ammonia, nitrite, and nitrate concentrations) were evaluated by one-way analysis of variance (ANOVA). Where ANOVA indicated significant overall effects (*p* < 0.05), pairwise comparisons were performed using Tukey’s Honest Significant Difference (HSD) post hoc test. For multivariate analyses, principal component analysis (PCA) was conducted in R (version 4.5.3) and was applied to the standardized (z-score) matrix of all quantitative probiotic and PGP parameters using the PCA function (FactoMineR package version 2.15). PCA biplots and scree plots were generated with fviz_pca_biplot and fviz_eig (factoextra package version 2.0.0). Hierarchical ascending classification (HAC) was performed on the same standardized dataset using Ward’s minimum variance agglomeration criterion. All plots were generated using ggplot2 version 4.0.3. Statistical significance was set at *p* < 0.05 throughout.

Calculation of the PGP index: Using R software version 4.6.0, a composite PGP index was computed for each isolate to summarize its overall plant growth-promoting potential across four quantitative traits: indole-3-acetic acid (IAA) production (µg/mL), siderophore production (SU, %), potassium-solubilization halo diameter (mm), and ammonia production (g/L). Because these traits were measured on different scales and in different units, each trait was first rescaled to a common 0 to 1 range by min–max normalization across the panel of 12 isolates, so that no single high-magnitude trait dominated the index. For each trait j and isolate i, the normalized value was calculated as

(7) X′ij=(Xij−Xj,min)÷(Xj,max−Xj,min)

where Xij is the mean value of trait j for isolate i (mean of replicate measurements), and Xj,min and Xj,max are the minimum and maximum values of that trait across all 12 isolates. This maps the lowest-performing isolate for a given trait to 0 and the highest to 1. The PGP index for each isolate was then defined as the arithmetic mean of its four normalized trait values (equal weighting), as follows:

(8) PGP index= (1/4) (X′IAA + X′Sid + X′K + X′NH3)

The resulting index is dimensionless and bounded between 0 and 1, where higher values indicate stronger cumulative PGP activity. Isolates scoring negative for a given trait (for example, isolates with no detectable IAA or siderophore production) contributed a normalized value of 0 for that component.

## 3. Results

### 3.1. Probiotic Traits

**Figure 1:**
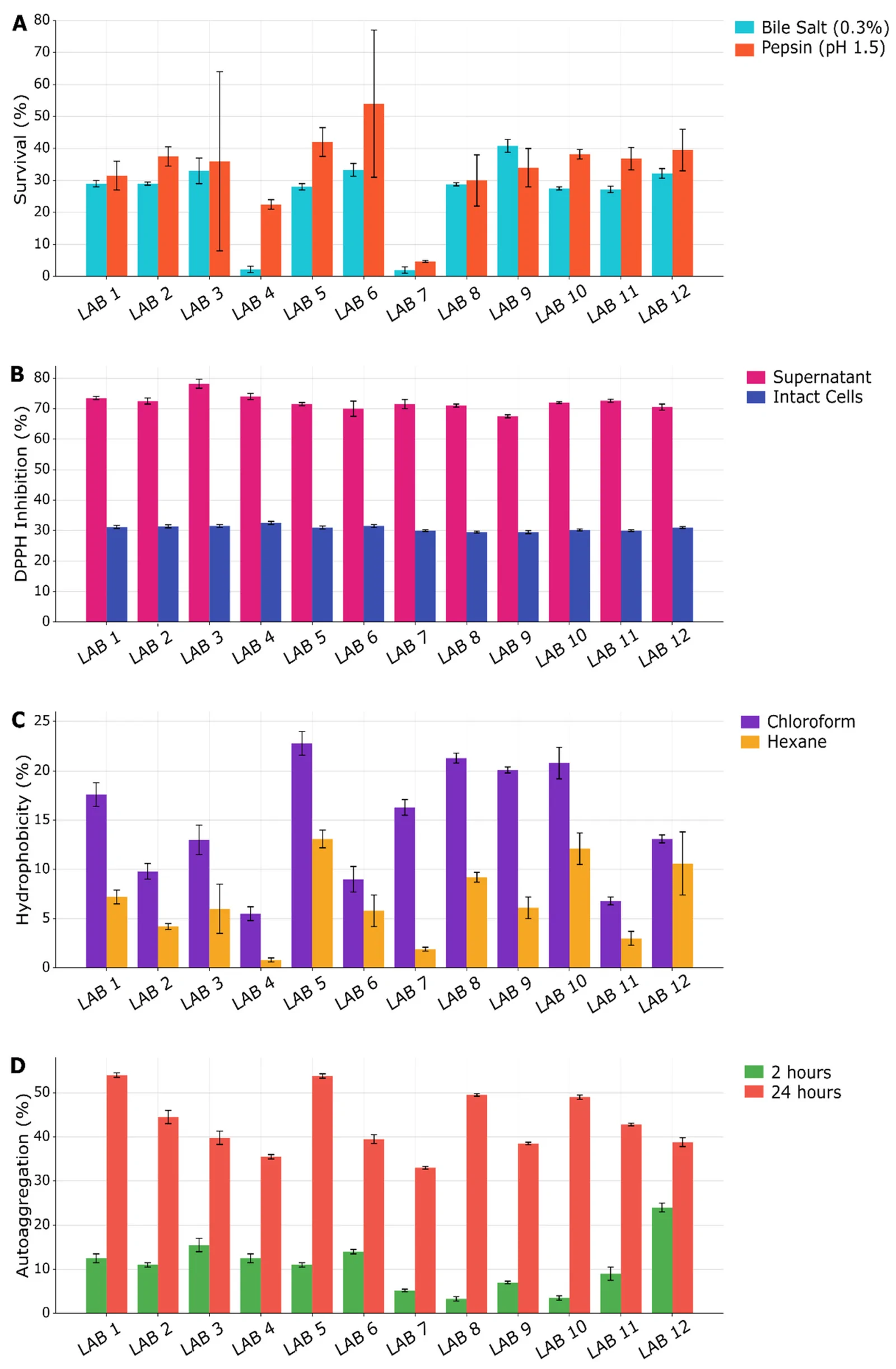
Functional characterization of LAB isolates under gastrointestinal stress, antioxidant capacity, cell surface properties, and auto-aggregation. (**A**) Survival (%) of LAB isolates (LAB 1–LAB 12) after exposure to 0.3% bile salts and pepsin (pH 1.5), indicating tolerance to simulated gastrointestinal conditions. (**B**) Antioxidant activity, measured as DPPH radical inhibition (%) in cell-free supernatant and intact cells. (**C**) Cell surface hydrophobicity (%), assessed using chloroform and hexane as solvents. (**D**) Auto-aggregation (%) of LAB isolates after 2 h and 24 h of incubation. Error bars represent standard deviations of triplicate measurements.

**Figure 2:**
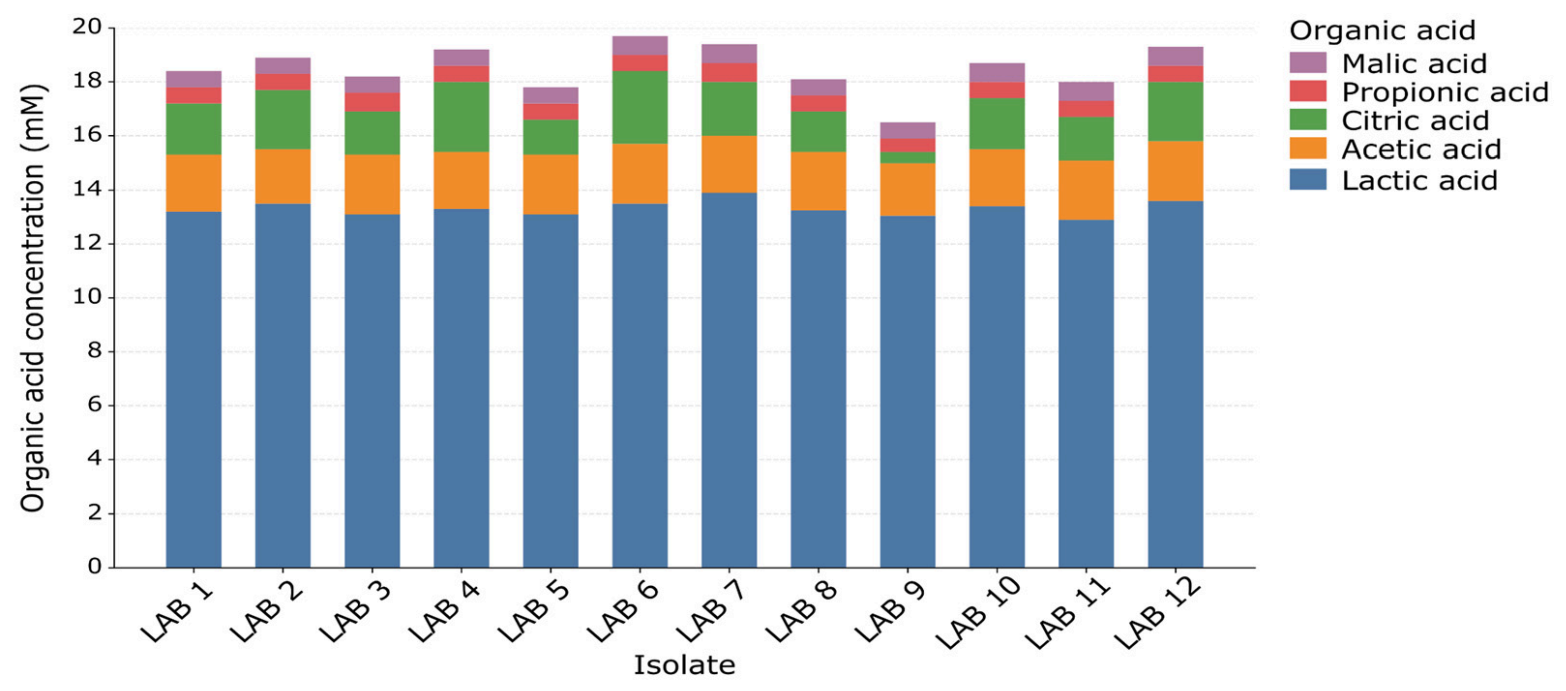
Stacked bar chart of organic acid production (mM) by the 12 LAB isolates.

Twelve LAB were isolated on MRS agar from tilapia (*Oreochromis niloticus*) farm environments within Nangui Abrogoua University (Abidjan) on the Ivory Coast (West Africa) and were characterized for their probiotic traits, including their microbial, metabolic, and safety profiles. The LAB isolates were assessed first for their antibacterial activities, illustrated in Figure S1, and then for their gastrointestinal tolerance and antioxidant and cell surface properties (Figure 1). Then, their capacity to produce organic acids (Figure 2) and enzymes (Table 1) was directly (e.g., chemical identification) or indirectly analyzed (e.g., activity measurement) using cell-free supernatant samples, and their safety was predicted via hemolysis and antibiotic sensitivity analysis (Table 2). The statistical analysis results and mean comparisons are supplied in the supplementary data (Data S1).

#### 3.1.1. Antibacterial Activity

All twelve LAB isolates demonstrated broad-spectrum antagonistic activity against four of the five indicator pathogenic bacteria tested. Strong inhibitory zones (6–17 mm) were observed against *E. coli* ATCC 25922 and *P. aeruginosa* ATCC 27853 across all isolates. Moderate inhibition (1–5 mm) was recorded against *S. aureus* ATCC 29213 and *A. hydrophila* LM 92844. No inhibition was observed against *V. cholerae* ATCC 7253 by any isolate under the assay conditions employed.

#### 3.1.2. Gastrointestinal Tolerance

An essential criterion in the selection of probiotic candidates is their ability to withstand conditions that simulate the gastrointestinal environment. The isolates showed varying tolerance among each strain to simulated gastrointestinal stressors (Figure 1A). Following 4 h exposure to 0.3% (*w*/*v*) bile salts, growth rates ranged from 2.13 ± 0.76% (LAB 7) to 40.87 ± 2.12% (LAB 9). Post hoc analysis revealed that LAB 9 displayed significantly higher bile tolerance than LAB 4 (mean difference = 38.62%, *p* < 0.0001) and LAB 7 (mean difference = 38.73%, *p* < 0.0001), whereas no significant differences were detected among most of the remaining isolates (*p* > 0.05).

Under simulated gastric conditions (0.3% pepsin, pH 1.5, 3 h), cell survival varied between 4.84 ± 0.26% (LAB 7) and 53.98 ± 13.28% (LAB 6). Statistical comparison confirmed that LAB 6 had significantly higher acid–pepsin resistance than LAB 7 (mean difference = 49.14%, *p* < 0.0001), LAB 4 (mean difference = 31.63%, *p* = 0.0012), and LAB 8 (mean difference = 23.92%, *p* = 0.0477). Similarly, LAB 5 showed a higher survival rate than LAB 7 (mean difference = 37.58%, *p* < 0.0001). In contrast, pairwise comparisons among the isolates with intermediate survival values (LAB 1 vs. LAB 8; LAB 2 vs. LAB 10) showed non-significant differences (*p* > 0.05). Overall, statistical analysis confirmed significant inter-strain differences for both bile salt tolerance and acid–pepsin resistance (*p* < 0.05). Except for LAB 4 and LAB 7, which were highly sensitive to both conditions, the remaining ten isolates preserved growth and survival rates within ranges widely regarded as acceptable for probiotic candidates (Figure 1A).

#### 3.1.3. Antioxidant Activity

Antioxidant activity is an important functional property in probiotic selection because it helps reduce oxidative stress by scavenging free radicals and reactive oxygen species. Probiotic strains with strong antioxidant potential may protect host cells from oxidative damage, contribute to gut health, and enhance overall health benefits. Therefore, the evaluation of antioxidant capacity is frequently included among the criteria for selecting promising probiotic candidates. DPPH radical-scavenging activity was measured for both intact cells and cell-free supernatants, for which the results are presented in Figure 1B. Intact cells showed moderate antioxidant capacity, with values ranging from 29.64 ± 0.11% (LAB 8) to 32.63 ± 0.00% (LAB 4). Post hoc analysis revealed that most pairwise comparisons among isolates were non-significant (*p* > 0.9999), indicating a broadly conserved antioxidant phenotype across the strains. However, LAB 12 displayed significantly higher scavenging activity than most other strains (*p* < 0.0001). In contrast, the cell-free supernatants exhibited higher scavenging activity (71–79%) across all isolates (Figure 1B), confirming that extracellular metabolites, rather than cellular components, constitute the primary source of antioxidant potential in these strains. The magnitude of this difference was >40 percentage points.

#### 3.1.4. Cell Surface Characteristics

Cell surface hydrophobicity is another property considered important for the probiotics’ overall adhesion capacity to various types of surfaces. Cell surface hydrophobicity was solvent-dependent, with consistently higher values observed using chloroform (5.50 ± 0.70% to 22.83 ± 1.17%) compared to hexane (0.83 ± 0.20% to 13.16 ± 1.17%), probably due to solvent polarity differences (Figure 1C). LAB 5 showed the highest hydrophobicity with both solvents; LAB 4 showed the lowest (Figure 1C). LAB 5 exhibited the highest hydrophobicity with both solvents, while LAB 4 showed the lowest. However, analysis of variance revealed that these differences were not statistically significant (*p* > 0.05).

Auto-aggregation is an important criterion in probiotic selection because it reflects the ability of bacterial cells to adhere to each other and form aggregates. A high auto-aggregation capacity may enhance the persistence of probiotics in the gastrointestinal tract, promote colonization of mucosal surfaces, and help prevent the attachment of pathogenic microorganisms by forming a protective barrier. Therefore, auto-aggregation is commonly evaluated as an indicator of the probiotic potential of bacterial strains. Auto-aggregation increased with incubation time for all strains (Figure 1D). After 2 h, values ranged from 3.06 ± 0.49% (LAB 8) to 23.87 ± 0.85% (LAB 12). Statistical comparison revealed that LAB 12 aggregated significantly more than LAB 1, LAB 2, and LAB 3 at this early point (*p* < 0.0001), indicating a rapid cell–cell adhesion phenotype unique to this strain. Following 24 h incubation, aggregation coefficients ranged from 32.50 ± 0.08% (LAB 7) to 52.89 ± 0.39% (LAB 1). Post hoc analysis confirmed highly significant inter-strain differences, with LAB 12 showing significantly lower aggregation than high-performing strains, for example, mean differences of −42 percentage points versus LAB 1, −48 versus LAB 3, and −58 versus LAB 5 (all *p* < 0.0001). Conversely, LAB 5 and LAB 1 clustered together at the upper extreme of the aggregation spectrum, with no significant difference between them (*p* > 0.9). Our finding indicates that while hydrophobicity is relatively conserved across isolates, auto-aggregation represents a highly strain-specific trait.

#### 3.1.5. Organic Acid Profile

High-performance liquid chromatography (HPLC) analysis of the cell-free supernatants confirmed the production of citric, malic, acetic, lactic, and propionic acid by all twelve LAB isolates. Succinic and butyric acids were not detected under the assay conditions employed. The overall metabolic profile remained uniform across the isolates. As expected, lactic acid was the major metabolite across all strains, with concentrations ranging from 12.93 to 13.96 mM. Acetic acid was produced at substantially lower levels (2.05–2.19 mM), followed by citric acid (0.29–2.72 mM), propionic acid (0.74–0.79 mM), and malic acid (0.44–0.54 mM) (Figure 2).

#### 3.1.6. Enzyme Production

Screening for extracellular hydrolytic enzymes revealed a consistent profile across the isolates (Table 1). None of the twelve strains produced detectable protease or lipase activity under the assay conditions. In contrast, amylase and cellulase production was observed in ten of the twelve isolates (LAB 1–5 and LAB 8–12), as evidenced by clear hydrolysis halos on starch- and carboxymethylcellulose-supplemented media, respectively. Strain-specific differences were confined to two isolates, i.e., LAB 6 and LAB 7, which failed to produce either amylase or cellulase.

#### 3.1.7. Safety Characteristics

Hemolysis and antibiotic sensitivity tests are commonly used for the first assessment of potential probiotic safety. All isolated LAB showed γ-hemolysis, i.e., they developed negative reactions to the hemolysis test performed on blood agar. Antibiotic susceptibility testing revealed varied resistance patterns across the twelve isolates (Table 2). All strains exhibited sensitivity to Ampicillin, Cefotaxime, Chloramphenicol, and Erythromycin, with inhibition zones exceeding 19 mm in most cases. Conversely, all isolates were generally resistant to Minocycline, Rifampicin, Ciprofloxacin, Penicillin, Gentamicin, Tetracycline, and Amikacin. Intermediate or strain-dependent resistance was recorded for Minocycline, Rifampicin, Penicillin, and Tetracycline. To quantify the cumulative burden of antimicrobial resistance, the Multiple Antibiotic Resistance (MAR) index was calculated for each isolate, defined as the ratio of antibiotics to which a strain exhibited non-susceptibility (intermediate resistance or full resistance) to the total number of antibiotics tested. MAR indices ranged from 0.500 to 0.583 across the collection (Table S1). Specifically, seven isolates (LAB 1, 2, 3, 4, 5, 6, 7, and 12) displayed resistance to six antibiotics (MAR = 0.500), whereas five isolates (LAB 8, 9, 10, 11, and 12) were non-susceptible to seven antibiotics (MAR = 0.583). The mean MAR index for the collection was 0.535 ± 0.035 (median = 0.500).

#### 3.1.8. Multivariate Analysis

**Figure 3:**
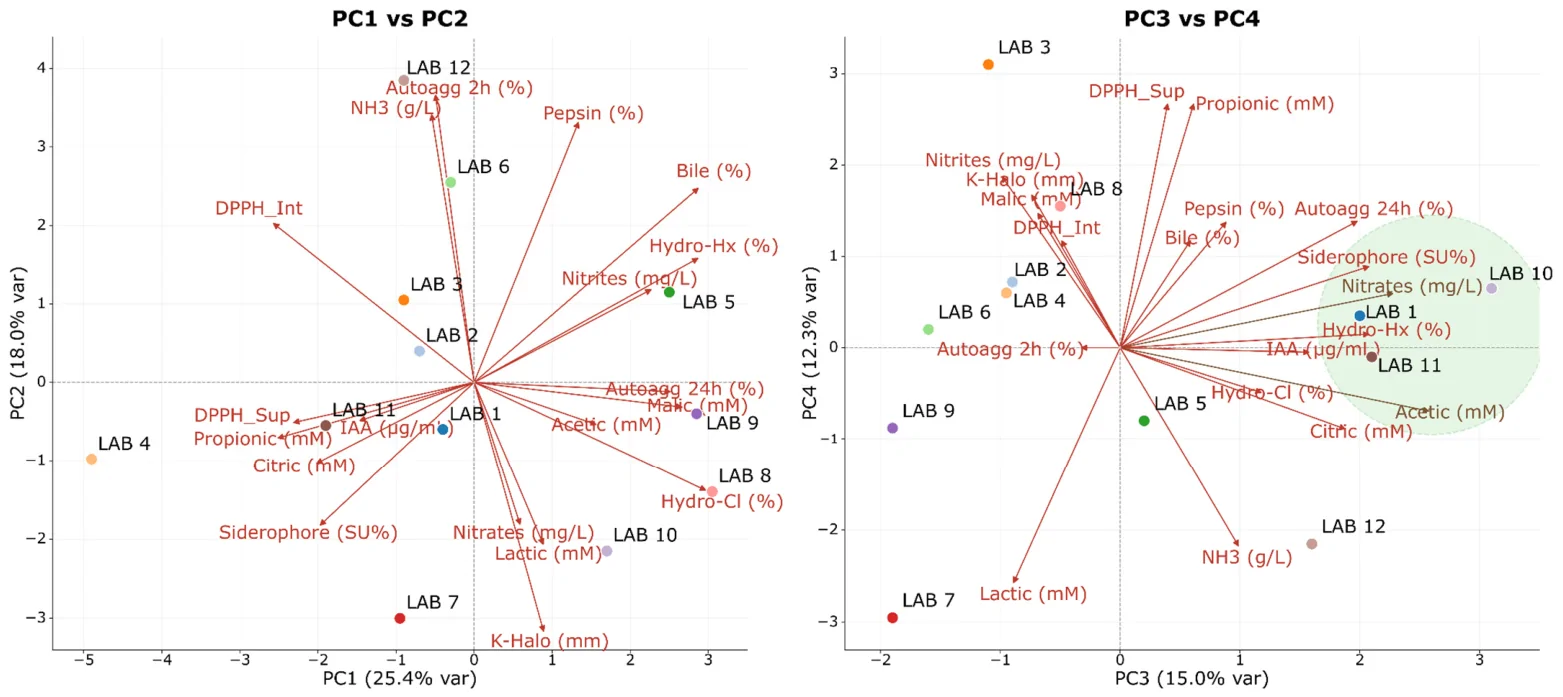
PCA biplot of 12 LAB isolates based on all quantitative traits. PC1 and PC2 account for 88.83% of total variance. Left panel: PC1 vs. PC2. Right panel: PC3 vs. PC4.

Principal component analysis (PCA), presented in Figure 3, was used to examine the multivariate relationships among all measured phenotypic, biochemical, and tolerance traits across the 12 LAB strains. The first four components accounted for 70.7% of the total variance (PC1: 25.4%; PC2: 18.0%; PC3: 15.0%; PC4: 12.3%). The PC1–PC2 biplot revealed a primary separation driven by gradients in organic acid production, nitrate/nitrite activity, surface hydrophobicity, bile and pepsin tolerance, auto-aggregation, and salt tolerance. The LAB strains positioned on the positive side of PC1 (LAB 8–10) were associated with stronger stress tolerance, higher acid production, and greater surface-associated properties, whereas strains on the negative side of PC1 (LAB 4 and LAB 7) aligned more closely with siderophore activity and citric/propionic associations but exhibited comparatively lower stress tolerance. PC2 was largely influenced by pepsin tolerance, bile tolerance, ammonia production, and antioxidant activity, with LAB 12 and LAB 6 showing strong associations with these traits. The PC3-PC4 biplot resolved secondary biochemical and antioxidant patterns. PC3 was associated with siderophore production, nitrate/nitrite levels, auto-aggregation, and hydrophobicity, separating strains such as LAB 10, LAB 1, and LAB 11.

### 3.2. Functional Properties

**Figure 4:**
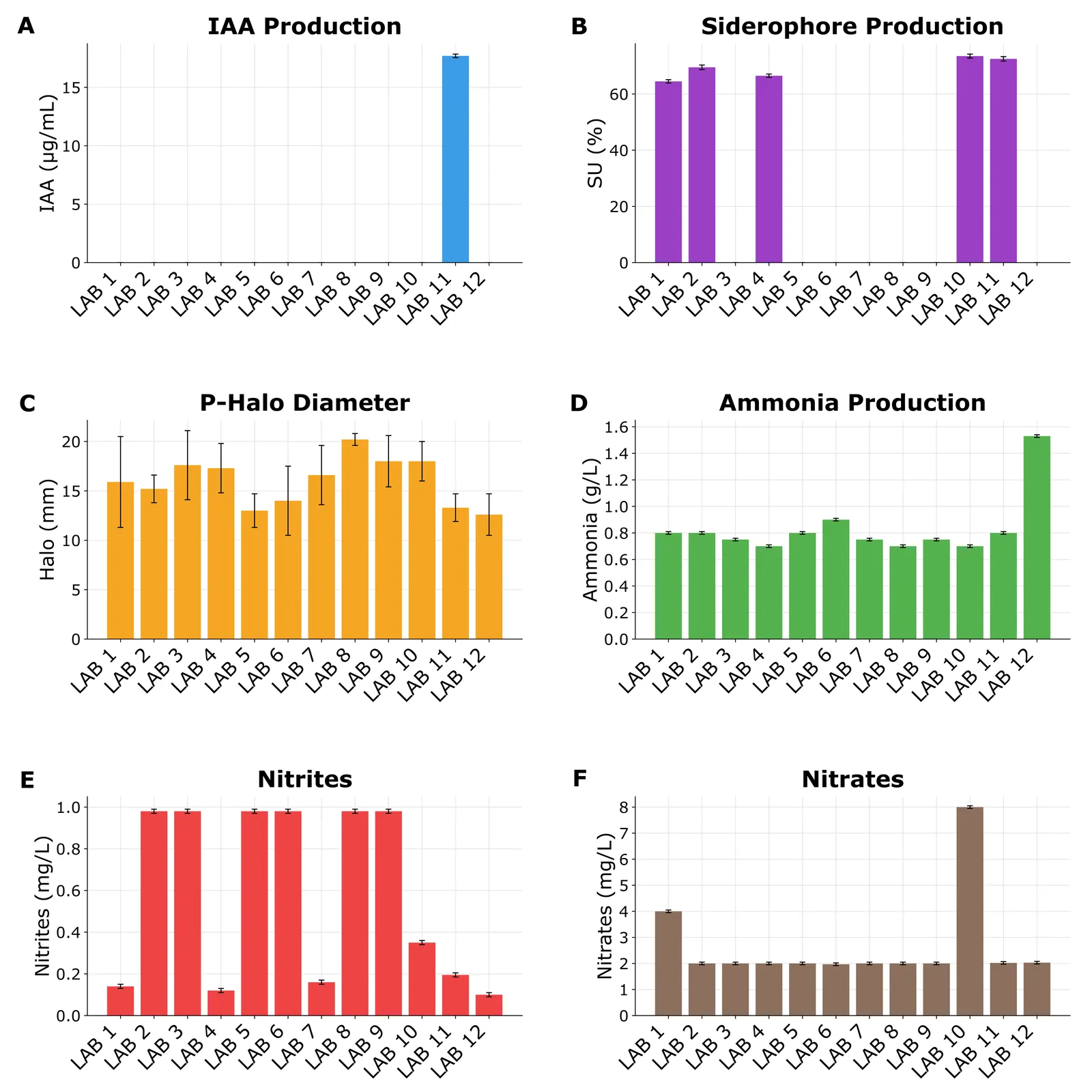
Bar charts showing PGP traits: (**A**) IAA production (µg/mL), (**B**) siderophore units (SU%), (**C**) phosphate-solubilization halo diameter (mm), (**D**) ammonia (g/L), (**E**) nitrites (mg/L), and (**F**) nitrates (mg/L) per strain (LAB 1–LAB 12). Values in (**A**,**D**–**F**) are final concentrations measured in cell-free supernatant after bacterial cultivation, with uninoculated medium used as blank. Error bars represent SD (*n* = 3).

A series of functional properties of the 12 LAB strains were characterized by individual PGP trait screening, which revealed a diverse distribution of functional capabilities and nitrifying capacities, as shown in Figure 4.

#### 3.2.1. Plant Growth-Promoting (PGP) Traits

Indole-3-acetic acid (IAA) production was observed exclusively in LAB 11, which yielded 17.72 ± 0.06 μg/mL (Figure 4A). Siderophore-mediated iron chelation was detected in five isolates (LAB 1, 2, 4, 10, and 11), with siderophore units (SU%) ranging from 64.45 ± 0.19% to 73.91 ± 0.56% (Figure 4B). Phosphate solubilization was a universal trait, observed in all twelve strains (clear halo diameters: 12.66 ± 2.08 to 20.33 ± 0.57 mm) (Figure 4C). Illustrations are presented in supplementary files (Figures S2–S4). Ammonium production was detected qualitatively and quantitatively in all LAB suspensions compared to the negative control (peptone water alone) after 4 days of incubation (Figure S5). The ammonium amount was significantly different among some strains (Figure 4D), varying from 0.7 to 1.54 g/L.

#### 3.2.2. Nitrifying Capacity

- In vitro efficiency

The LAB efficiency in nitrite and nitrate production can be categorized into three main groups, depending on the strain, as shown in Figure 4E,F. Numerical data are provided in Table S2. While some strains, namely LAB 2, 3, 5, 6, 8, 9, accumulated the highest, similar nitrite concentrations around 1,0 mg/L (Figure 4E), LAB 10 showed the strongest nitrifying phenotype, accumulating the highest concentration in nitrates (8 mg/L), followed by LAB1 (4 mg/mL), with the others providing the lowest, similar level (2 mg/L) (Figure 4F). Accordingly, two strains (LAB10 and LAB1) among the twelve showed the maximal conversion of ammonia to oxidized forms and were tested in vivo using trout pond water.

b. In vivo assessment

**Figure 5:**
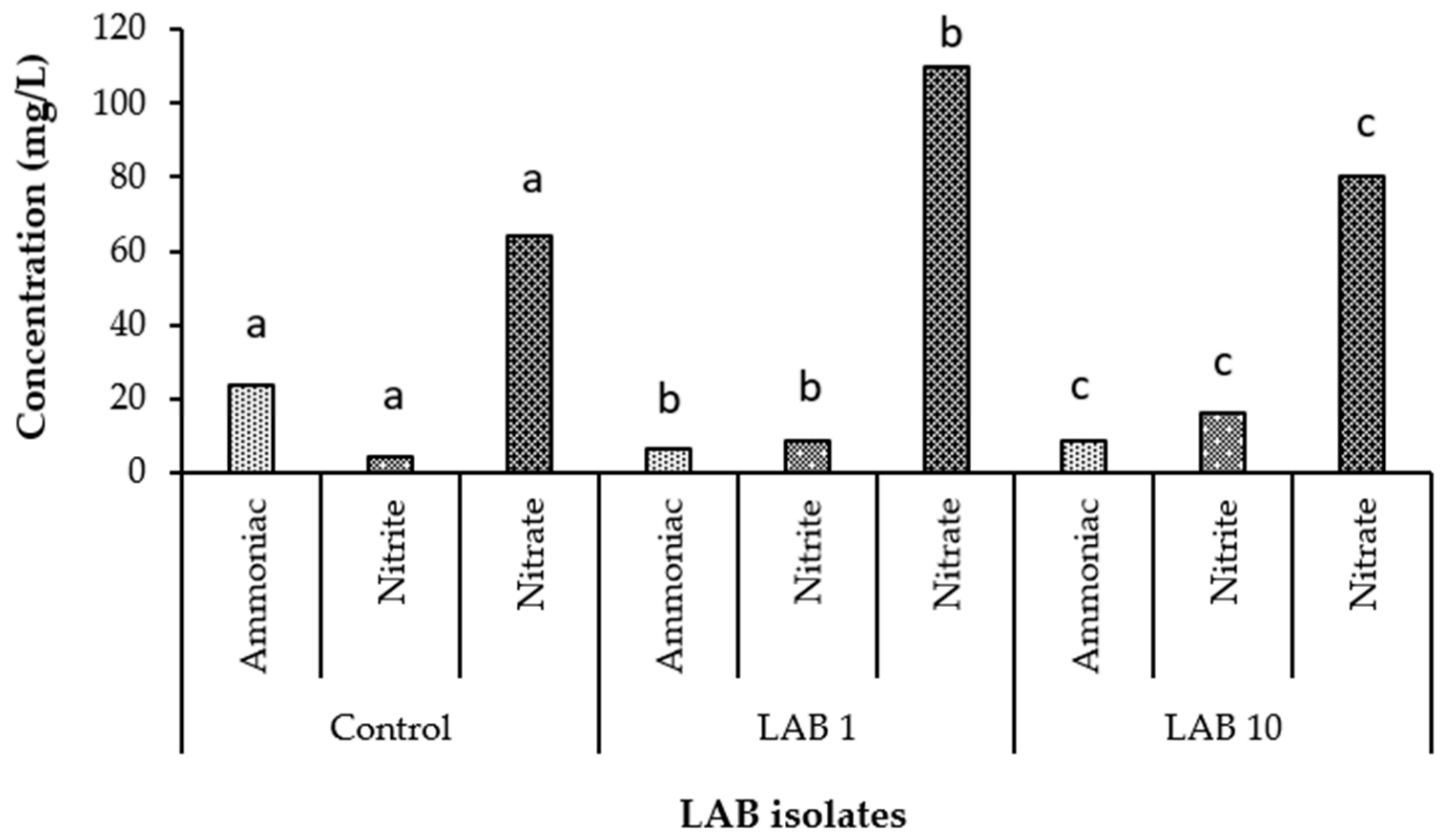
Ammonia, nitrite and nitrate concentrations measured after 7 days of trout pond water inoculation by LAB1 and LAB 10 compared to uninoculated sample (negative control) without water exchange. Each concentration (mg/L) represents a triplicate average and is significantly different (*p* < 0.05) to others, as indicated by a, b, c.

Ammonia, nitrite, and nitrate content of inoculated and uninoculated (negative control) trout pond water samples with LAB1 and LAB10 are shown in Figure 5. LAB 10 inoculation yielded higher ammonia (8.8 mg/L) and nitrite (16 mg/L) levels relative to LAB 1 (6.4 and 8.8 mg/L, respectively), whereas LAB 1 demonstrated superior complete nitrification, producing significantly higher nitrate concentrations (110 mg/L compared to 80 mg/L for LAB 10).

#### 3.2.3. Composite Index

**Figure 6:**
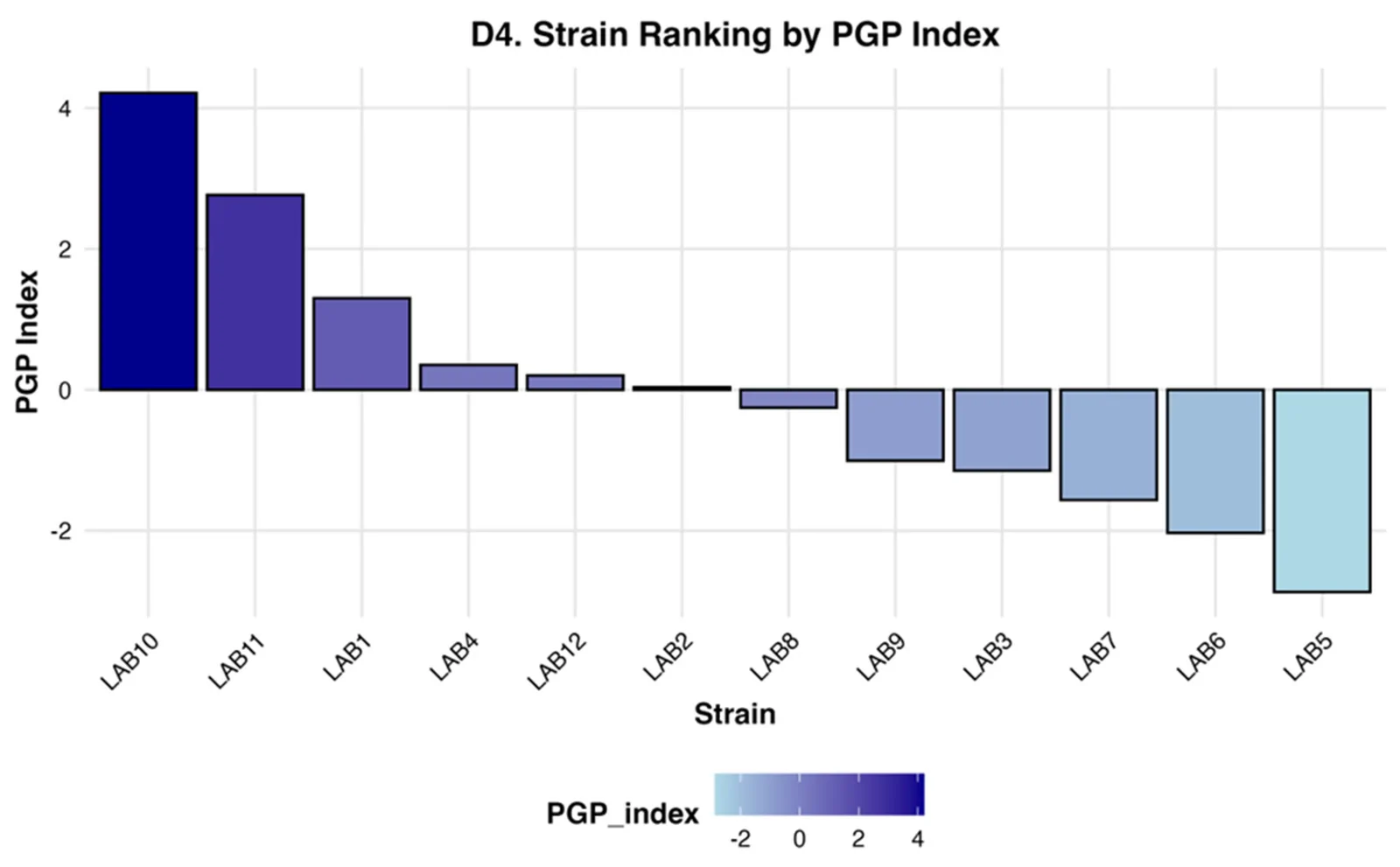
Strain ranking based on the composite plant growth-promoting (PGP) index. Positive values indicate stronger overall expression of plant growth-promoting traits, whereas negative values reflect comparatively weaker combined performance.

To synthesize these multifaceted traits into a single evaluative metric, a composite PGP index was calculated and ranked (Figure 6). The index stratified the isolates into a clear performance hierarchy: LAB 10 and LAB 11 emerged as the top-performing strains (PGP Index > 2.5), driven by their combined siderophore production, universal phosphate solubilization, and (in the case of LAB 11) exclusive IAA synthesis. LAB 1 ranked third (Index = 1.3), followed by LAB 4 and LAB 12, with moderate positive scores. The remaining isolates (LAB 2, 8, 9, 3, 7, 6, and 5) exhibited near-zero or negative index values. This composite ranking corroborates the individual trait data and informs the selection of LAB 1, LAB 10, and LAB 11 as lead candidates for aquaponic bioaugmentation.

#### 3.2.4. Maize Seed Germination

**Figure 7:**
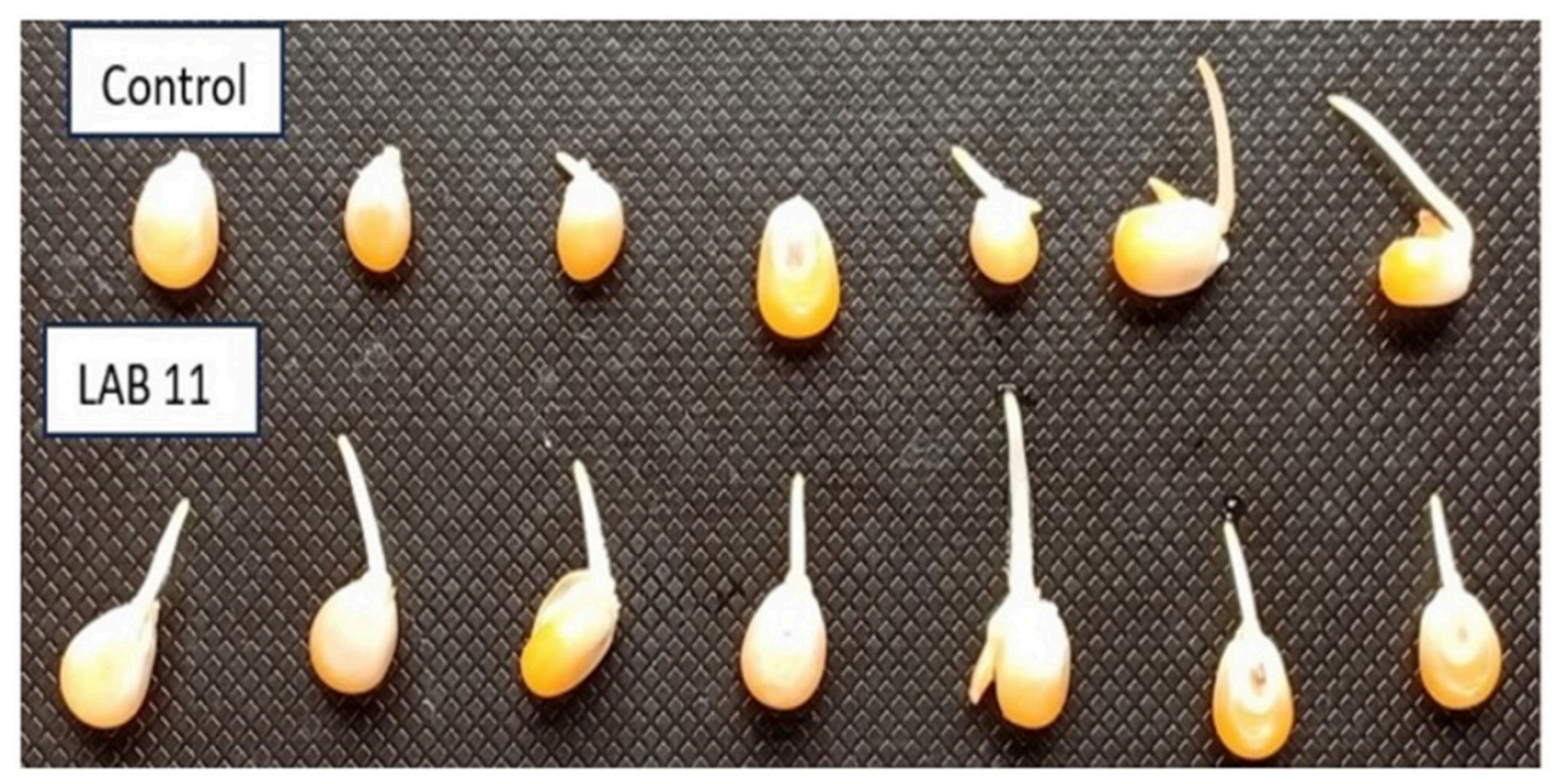
Maize germination among seeds treated with LAB11 supernatant vs. negative control (water).

A difference in germination between the LAB 11 isolate and control was observed (Figure 7), showing the germination rate (%) and the radicle length (mm) of the germinated maize seeds. The germination rate was highest with the LAB 11 isolate (86.66 ± 5.77%) compared to the control (63.33 ± 32.14%). Moreover, radicle length (mm) was also greater with the LAB 11 isolate (7 ± 0.52 mm) than the control (4.94 ± 1.52 mm). As such, a significant difference (*p* < 0.05) was observed between LAB 11 and the control.

### 3.3. Molecular Characterization of LAB Strains

**Figure 8:**
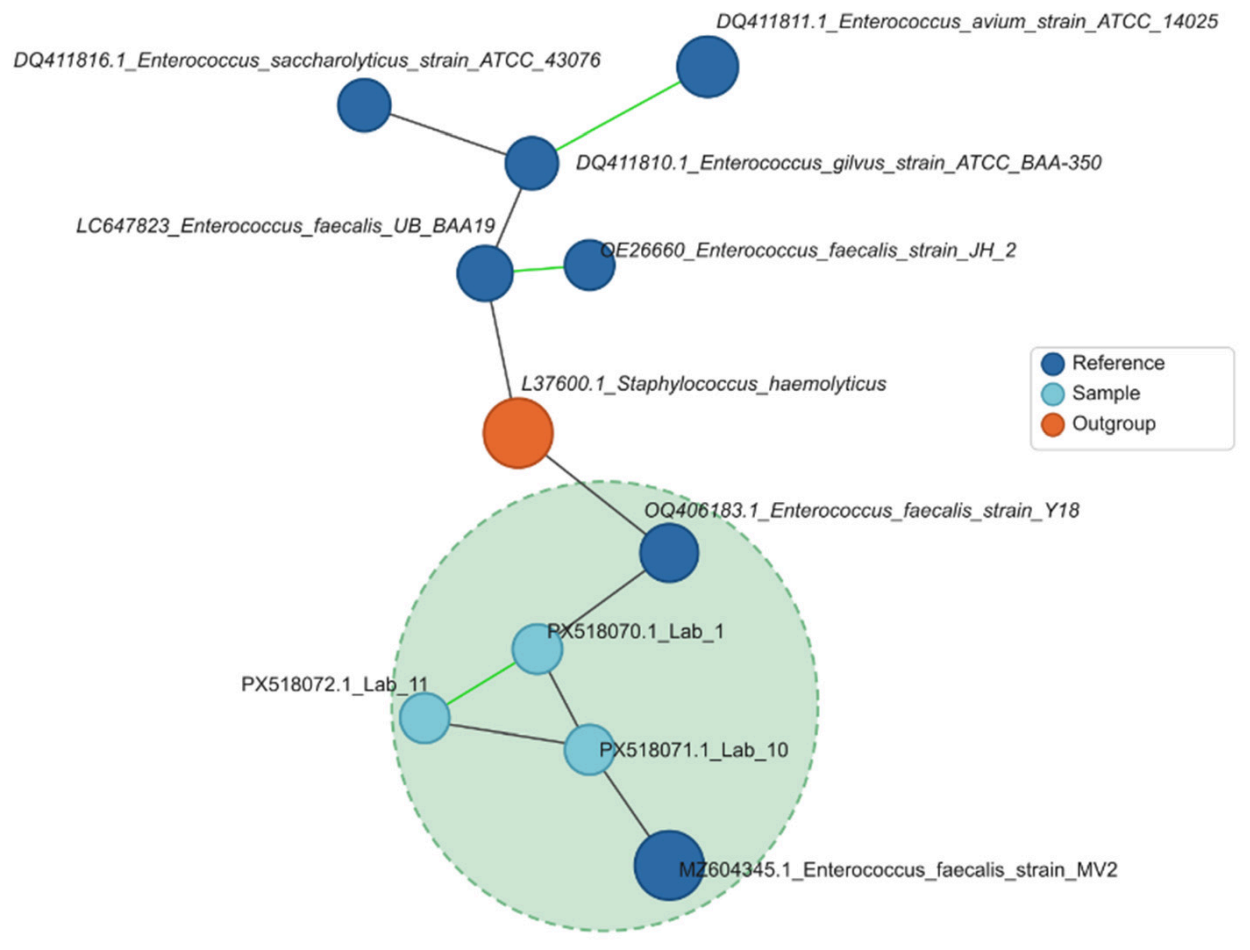
Neighbor-joining phylogenetic tree based on 16S rRNA gene sequences and whole genome sequence comparisons of the isolates and closely related reference strains. It shows the evolutionary relationships of the three selected LAB strains with NCBI reference strains and an outgroup. LAB 1 (PX518070), LAB 10 (PX518071), and LAB 11 (PX518072) cluster tightly within a distinct *Enterococcus faecalis* clade (highlighted in green), exhibiting closest phylogenetic affinity to reference *E. faecalis* strains Y18 and MV2. *Staphylococcus haemolyticus* was used as the outgroup to root the tree. Branch lengths are proportional to genetic distance.

Phylogenetic analysis based on 16S rRNA gene sequences confirmed that LAB 1, LAB 10, and LAB 11 belonged to *Enterococcus* genera. However, because 16S sequences alone are insufficient to resolve fine-scale genomic relationships due to their extreme conservation, we constructed an NLV graph based on whole genome sequence comparisons of our isolates and closely related reference strains (Figure 8). The neighbor-joining phylogenetic tree, rooted with *Staphylococcus haemolyticus* (L37600.1) as the outgroup, revealed that all three isolates cluster tightly within a monophyletic *E. faecalis* clade (Figure 8). This clade includes the reference strains *E. faecalis* Y18 (OQ406183.1) and *E. faecalis* MV2 (MZ904345.1), with the study isolates forming a distinct subcluster supported by bootstrap analysis. The three isolates are clearly delineated from other *Enterococcus* species, including *E. avium* (DQ411811.1), *E. saccharolyticus* (DQ411816.1), and *E. gilvus* (DQ411810.1), confirming their species-level assignment.

## 4. Discussion

This study provides a comprehensive multi-trait evaluation of LAB isolated from a West African tilapia farm environment covering probiotic, PGP, and nitrifying properties simultaneously, a combination rarely addressed in a single study. This multi-dimensional approach was used in light of recent calls for innovative research to unearth new probiotic strains capable of sustaining the expanding aquaculture industry while reducing antibiotic dependency [6]. Rahayu et al. [6] emphasized that among the most promising probiotic groups in aquaculture are LAB that improve digestion, modulate immunity, and enhance water microbial composition, functions that can be uniquely amplified when these bacteria also possess plant growth-promoting and nitrifying traits. Here, we identify three *E. faecalis* strains (LAB 1, LAB 10, LAB 11) that have a favorable-safety genomic profile, probiotic characteristics [28], and unique potential for integrated aquaponic bioaugmentation.

The broad-spectrum antibacterial activity of the isolates, with inhibition of *E. coli, P. aeruginosa, A. hydrophila*, and *S. aureus* but not *V. cholerae*, aligns with published findings for *Enterococcus* spp. and related LAB from aquaculture environments [39,48,49]. The abundant organic acids documented in our study, particularly lactic acid and acetic acid, likely contribute mechanistically to the observed antibacterial activities, mainly against *Aeromonas hydrophila*, which represents a key pathogen in tilapia and prawn aquaculture. The inability to inhibit *V. cholerae* under the conditions tested may reflect the specific antimicrobial compounds produced (organic acids) and their activity spectra [50]. Mechanistically, LAB exert antibacterial effects through multiple complementary pathways, including competitive exclusion for adhesion sites, secretion of organic acids that reduce pH and bacteriocins that directly inhibit pathogen growth, enhancement of intestinal barrier function, and immunomodulation of the mucosal immune response [24].

Our findings, along with reports in other studies, suggest that the inhibition spectra of *Enterococcus* CFSs against plant pathogenic bacteria varied [24] depending on the precise composition and concentration of the produced metabolites relative to species-specific minimum inhibitory thresholds. Notwithstanding, the CFS from our LAB isolates demonstrated an impressive inhibitory range, effectively suppressing four of five clinically and aquaculturally relevant pathogens.

GI tolerance data confirm that most isolates, except for LAB 4 and LAB 7, can withstand conditions simulating the fish digestive tract with survival rates exceeding those reported by Coulibaly et al. [29] for LAB from tilapia intestines under pepsin/acid pH (up to 53.98%). A comparative analysis on *E. faecium* MYSBC14 from blue cherry fruit under analogous conditions revealed a cell survival rate of 38.08% at pH 2 after 2 h (declining to 23.16% at 4 h) and bile salt (0.3% oxgall) tolerance of 52.67% after 2 h (43.01% at 4 h) [24]. The performance of the present isolates at the more stringent pH 1.5/pepsin condition therefore compares favorably with well-characterized *Enterococcus* probiotic candidates from diverse ecological niches. The DPPH scavenging data, with supernatant activities reaching 78.4%, are consistent with the production of antioxidant compounds and compare favorably with values reported for LAB from other sources [51,52,53]. This antioxidant capacity is of direct relevance to fish welfare, as oxidative stress is a key mediator of inflammation and immunosuppression under intensive aquaculture conditions.

The cell-surface analysis revealed that hydrophobicity values were solvent-dependent, with chloroform yielding higher values than hexane for all strains, consistent with the literature [54]. LAB 5 showed the highest hydrophobicity and values among the highest 24 h auto-aggregations (52.82%), supporting a positive interdependency between these properties [54,55], even though other surface mechanisms, e.g., electrostatic interactions, may also drive aggregation independently of hydrophobicity [55].

The dominance of amylase and cellulase production among 10 of the 12 isolates, as well as the complete absence of protease and lipase activity, is a specific profile that may actually be advantageous for modern tilapia farming, where commercial feeds are increasingly formulated with plant-derived ingredients such as soybean, corn, and wheat to reduce costs and environmental impact. These plant materials contain high levels of starches and complex fibers that fish cannot fully digest on their own, leading to poor nutrient absorption and increased organic waste. By producing abundant amylase and cellulase, these LAB strains effectively act as natural digestive aids, breaking down tough plant carbohydrates into simpler sugars that the fish can readily absorb. This enhances feed conversion efficiency, supports healthier growth, and significantly reduces undigested waste that would otherwise degrade water quality. Furthermore, the lack of protease and lipase activity is not a limitation but rather a practical benefit. Tilapia naturally produces sufficient protein- and fat-digesting enzymes, and excessive bacterial proteolysis could prematurely degrade valuable dietary proteins or generate excess nitrogenous waste, both of which could compromise fish health and system stability. The enzyme profile detected in this study differs from that reported for gut-derived LAB [29,56], which more commonly produce lipase and β-galactosidase.

All isolates were non-hemolytic and sensitive to the key clinically relevant antibiotics Ampicillin and Chloramphenicol, satisfying two primary probiotic safety criteria [57,58]. The intrinsic resistances observed (Rifampicin, Ciprofloxacin, Penicillin, Gentamicin, Tetracycline, Amikacin) are well documented for *Enterococcus* spp. and are considered non-transferable when chromosomally encoded. However, transferability assessment via molecular methods is recommended before regulatory approval [59,60,61].

Among PGP traits, LAB 11 stood out as the sole IAA producer (17.72 µg/mL), confirming a dose within the stimulatory range that promotes root development and seed germination [62,63,64]. This value exceeds the 11.56 µg/mL reported for *E. faecium* MYSBC14 [24]. This result is comparable to the IAA outputs documented for *Enterococcus* sp. SR9 by Batool et al. [25], who selected this strain for its auxin-producing capacity to synthesize silver nanobiofertilizers. The significant improvement in maize germination rate (+23 percentage points) and radicle length (+2.06 mm) observed relative to the control provides direct phenotypic evidence of bio-stimulant activity, consistent with reports for *L. plantarum* on tomato [65] and *E. faecium* on cherry [24]. Batool et al. [25] similarly demonstrated that wheat seeds treated with *Enterococcus* sp. SR9 achieved 98% germination and a vigor index of 1928, the highest among all bacterial isolates tested, showing that auxin-producing *Enterococcus* strains can deliver robust phytostimulatory effects across multiple crop species. For upland rice, Cavite et al. [66] reported that inoculation with the multi-trait PGPR IBBw1a increased root length by 60% and vigor index by 53% relative to uninoculated controls. The bio-stimulant effect of LAB 11 CFS on maize, while measured at 1:50 dilution, is therefore mechanistically plausible and agronomically relevant. The siderophore production by LAB 1, 2, 4, 10, and 11 further extends their PGP potential through iron chelation and mobilization, which can limit the proliferation of iron-dependent pathogens in the rhizosphere while simultaneously increasing iron bioavailability for the plant [67]. Universal phosphate solubilization across all twelve isolates was likely mediated by organic acid secretion [68]. Lactic acid, the major metabolite produced by the present isolates, has been specifically linked in the dissolution of insoluble calcium phosphates at low temperatures by Yan et al. [69], who further documented that citric acid secretion by *Rahnella aquatilis* K-1 drove potassium solubilization (23.02 mg/L K) from mineral silicates. The organic acid profiles documented for the present strains (lactic, acetic, citric, malic, and propionic acids) are therefore mechanistically well suited for both P and K solubilization in aquaponic and hydroponic matrices. This multi-trait PGP character in *Enterococcus* isolates has also been exploited in agricultural biotechnology. Li et al. [26] incorporated LAB into a Se-enriched biochar fertilizer that enhanced tomato chlorophyll content by 85.6% and enriched beneficial rhizosphere taxa, demonstrating that LAB-based biofertilizers can positively reshape soil and rhizosphere microbiome composition, an effect equally relevant in aquaponic biofilm communities.

The nitrifying capacity of LAB 1 and LAB 10, demonstrated both in vitro and in trout pond water, is a particularly novel finding. In aquaponic systems, ammonia from fish metabolism is toxic at elevated concentrations and must be converted through the nitrogen cycle to provide nitrate for plant nutrition. LAB strains capable of ammonia conversion could therefore serve dual roles, first contributing to biological water treatment, and second, supplying nitrogen to plants, like the traditional role of nitrifying bacteria such as *Nitrosomonas* spp., but in a probiotic context. The production of nitrite and nitrate by the LAB isolates observed in this study suggests the occurrence of strain-dependent heterotrophic nitrification-like processes, as discussed in detail on the LAB1, LAB10, and LAB11 whole genome analysis related to their nitrifying capacity [28]. Although LAB are not traditionally recognized as classical nitrifying microorganisms, some strains may contribute to ammonia oxidation through alternative metabolic pathways involving oxidoreductase activities and the generation of reactive oxygen intermediates. The significant differences observed between the two strains producing the greatest nitrate content, LAB 10 and LAB 1, suggest distinct capacities for nitrogen oxidation. LAB 10 accumulated greater amounts of nitrite than LAB1, suggesting a stronger ammonia oxidation activity with LAB10 and a more efficient conversion of nitrite to nitrate with LAB1, which was also observed in vivo using the pond-water microcosm. These observations may reflect differences in enzymatic activities, oxygen utilization, redox metabolism, or other yet unidentified nitrogen-transforming mechanisms. The ability of certain LAB strains to transform toxic ammonia into less harmful oxidized nitrogen species could represent an additional probiotic trait contributing to nitrogen cycling and water quality improvement in aquaculture systems.

Growth of the LAB isolates in the ammonia-oxidizing bacteria culture medium was unexpected because LAB are generally regarded as chemoorganotrophic microorganisms that rely on organic substrates as sources of carbon and energy. The observed growth may indicate the ability of these strains to survive and maintain metabolic activity under nutrient-limited conditions through the utilization of trace organic compounds, inoculum-derived nutrients, or endogenous cellular reserves. Concurrent nitrite and nitrate production suggests that the strains may perform heterotrophic nitrification-like processes, in which ammonia is oxidized while organic carbon supports cell maintenance and growth. Unlike classical autotrophic ammonia oxidizers that derive energy directly from ammonia oxidation, LAB may employ alternative oxidoreductase systems and intracellular redox reactions contributing to nitrogen transformation. These findings suggest an underexplored role of LAB in nitrogen cycling and warrant further investigations to elucidate the genetic and enzymatic basis for ammonia transformation in these strains. Given that canonical ammonia-oxidizing and nitrite-oxidizing pathways have not been well documented in LAB, further genomic and enzymatic investigations are required to elucidate the molecular basis of ammonia oxidation and nitrate formation in these strains.

The functional significance of ammonia-oxidizing bacteria in crop rhizospheres has been established through qPCR quantification of the amoA gene, which encodes the alpha subunit of ammonia monooxygenase, the key enzyme of canonical autotrophic nitrification. Ke et al. [70] demonstrated that inoculation of maize with the diazotrophic endophyte *Pseudomonas stutzeri* A1501 significantly increased the abundance of ammonia-oxidizing bacteria (amoA copy numbers per g dry soil) and enhanced plant nitrogen accumulation by 0.30–0.82 g N/plant, depending on water regime, thereby directly linking bacterial nitrogen cycling genes to plant nutritional outcomes. Independently, Di Benedetto et al. [27] included nitrification as one of four primary quantitative screening criteria for PGPB selection from the durum wheat rhizosphere, alongside siderophore production, phosphate solubilization, and ammonium production, stressing the agronomic relevance of nitrification capacity as a PGPB trait.

The identification of LAB 1, LAB 10, and LAB 11 as *E. faecalis* is particularly significant. While some *Enterococcus faecalis* strains carry virulence factors, the absence of hemolysis and the favorable antibiotic profile observed here are important positive indicators. In addition, full gene screening indicated a favorable safety profile in terms of absent cytolysin and vancomycin resistance, as well as a strong genomic signature characteristic of recognized probiotics with high probability scores (0.80–1.00) obtained using ProbML [28].

All these data suggest that a defined consortium of LAB 1 + LAB 10 + LAB 11 could collectively provide complementary probiotic benefits for tilapia GI health and immune modulation; promote plant growth through IAA, siderophores, and phosphate solubilization; and support biological nitrification. This multifunctional consortium approach is similar to the multi-trait PGPB selection strategy advocated by Di Benedetto et al. [27]. These authors demonstrated that combining three isolates (6P, 20P, 25A) with complementary functional traits, such as phosphate solubilization, siderophore production, nitrification, and IAA biosynthesis, yielded the greatest overall plant growth benefit (up to 50% increase in biomass and 25% increase in height in durum wheat) for consortium application. In our study, it can be concluded that LAB 1 and LAB 10 provide nitrification capacity and siderophore production, while LAB 11 uniquely contributes to IAA biosynthesis and strong germination bio-stimulant activity. Furthermore, the broader capacity of LAB-based biofertilizers to positively modulate rhizosphere microbiome composition, as demonstrated by Li et al. [26] through the enrichment of beneficial taxa, including *Paracoccus, Stutzerimonas*, and *Streptomyces*, after LAB biochar fertilizer application in tomato, suggests that the proposed consortium could confer secondary ecosystem services by reshaping the aquaponic biofilm community in favor of plant-beneficial and water-quality-improving microorganisms. This multifunctional consortium approach represents a promising and novel strategy for integrated aquaponic bioaugmentation, complementing existing biofilter microbiomes. Consortium stability and inter-strain competitive interactions under real aquaponic conditions will require dedicated co-culture and mesocosm studies to validate the synergistic effects predicted from individual strain characterization.

## 5. Conclusions

In conclusion, the screening of the probiotic and functional properties of twelve LAB isolates from a tilapia farm environment on the Ivory Coast led to the selection of three promising strains (LAB 1, LAB 10, and LAB 11), all identified as *Enterococcus faecalis*. In addition to exhibiting antibacterial and antioxidant activities, GI tolerance, favorable cell-surface properties, and enzymatic production capacities, these isolates uniquely combine probiotic characteristics with plant growth-promoting (PGP) and nitrifying abilities. Notably, LAB 11 significantly enhanced maize seed germination and radicle growth, confirming its bio-stimulant potential.

Collectively, these three *E. faecalis* strains represent promising candidates for a microbial consortium, owing to their probiotic potential, favorable safety profiles, and complementary multifunctional properties. Their combined attributes make them particularly suitable for dual-purpose applications, such as improving both fish and plant performance in aquaponic systems.

Future studies should focus on in vivo co-culture trials using these selected strains in a tilapia–plant aquaponic model, as well as on evaluating consortium stability, persistence, and competitive fitness during long-term cultivation. Moreover, further comprehensive safety assessments, including in vivo studies, are warranted to substantiate the safety of these strains and exclude potential risks to human health prior to any applied use. Such investigations will be essential to validate their efficacy and practical applicability under aquaponic production conditions.

## Figures and Tables

**Table 1:** Enzymatic profile of 12 LAB isolates. (+) indicates production; (−) indicates absence.

Strain	Protease	Lipase	Amylase	Cellulase
LAB 1	−	−	+	+
LAB 2	−	−	+	+
LAB 3	−	−	+	+
LAB 4	−	−	+	+
LAB 5	−	−	+	+
LAB 6	−	−	−	−
LAB 7	−	−	−	−
LAB 8	−	−	+	+
LAB 9	−	−	+	+
LAB 10	−	−	+	+
LAB 11	−	−	+	+
LAB 12	−	−	+	+

**Table 2:** Antibiotic susceptibility profiles of LAB isolates.

	MIN	AM	RA	AUG	C	CIP	TET	CTX	GEN	P	E	AN	S	IR	R
LAB 1	22S	25S	16IR	30S	30S	0R	14R	21S	0R	20S	24S	0R	7	1	4
LAB 2	17IR	32S	16IR	25S	20S	0R	22S	21S	0R	15IR	20S	0R	6	3	3
LAB 3	16IR	20S	20S	35S	30S	0R	15R	23S	0R	14R	25S	0R	6	1	5
LAB 4	15IR	40S	30S	33S	30S	0R	18IR	35S	0R	15IR	30S	0R	6	3	3
LAB 5	20S	32S	18IR	35S	27S	0R	14R	23S	0R	14R	26S	0R	6	1	5
LAB 6	18IR	29S	20S	35S	34S	0R	13R	23S	0R	13R	22S	0R	6	1	5
LAB 7	18IR	25S	20S	28S	30S	0R	13R	24S	0R	17IR	24S	0R	6	2	4
LAB 8	18IR	25S	0R	30S	20S	0R	11R	21S	0R	10R	20S	0R	5	1	6
LAB 9	12R	30S	0R	26S	27S	0R	13R	20S	0R	12R	25S	0R	5	0	7
LAB 10	18IR	35S	18IR	34S	27S	0R	15R	25S	0R	16IR	21S	0R	5	3	4
LAB 11	15R	35S	19IR	25S	25S	0R	15R	21S	0R	16IR	21S	0R	5	2	5
LAB 12	15R	34S	20S	30S	28S	0R	14R	21S	0R	16IR	21S	0R	6	1	5

Resistant (R) ≤ 14 mm; intermediate resistance (IR) 15–19 mm; sensitive (S) > 19 mm. Minocycline (MIN): 30 µg; Tetracycline (TET): 30 µg; Ampicillin (AM): 10 µg; Cefotaxime (CTX): 5 µg; Penicillin (P): 1 µg; Amoxicillin + Clavulanic acid (AUG): 30 µg; Rifampicin (RA): 5 µg; Chloramphenicol (C): 30 µg; Ciprofloxacin (CIP): 5 µg; Gentamicin (GEN): 10 µg; Erythromycin (E): 15 µg; Amikacin (AN): 30 µg.

## Data Availability

The 16S rRNA gene sequences are deposited under GenBank accessions PX518070 (LAB1), PX518071 (LAB10), and PX518072 (LAB11). The draft genome sequences of LAB1, LAB10, and LAB11 are deposited in the NCBI GenBank database under BioProject PRJNA1453999. The BioSample accessions are SAMN57287360 (LAB1), SAMN57287361 (LAB10), and SAMN57287362 (LAB11). The Whole Genome Shotgun (WGS) project accession numbers are JBXPLQ000000000 (LAB1), JBXPLP000000000 (LAB10), and JBXPLO000000000 (LAB11). Raw sequencing reads are available through the same BioProject (PRJNA1453999) via the NCBI Sequence Read Archive. Further inquiries can be directed to the corresponding author.

## Supplementary Materials

The following supporting information can be downloaded at https://www.mdpi.com/article/10.3390/microorganisms14091906/s1, Figure S1: Illustration of the antibacterial activity agar plate of the 12 LAB isolates against *Pseudomonas aeruginosa* determined by well-diffusion assay; Figure S2: Indole-3-acetic acid (IAA) production was observed exclusively in LAB 11; Figure S3: Representative agar plate showing siderophore production by LAB isolates; Figure S4: Representative *Phosphate solubilization* activity of LAB strain; Figure S5: Representative ammonia production activity; Table S1: Antibiotic resistance profile and multiple antibiotic resistance (MAR) index across 12 LAB strains. Table S2: Optical density, ammonia, nitrite, and nitrate concentrations from the in vitro nitrification assay.

## Abbreviations

**Abbreviation**	**Definition**
**CFS**	Cell-free supernatant
**DPPH**	2,2-diphenyl-1-picrylhydrazyl
**EFSA**	European Food Safety Authority
**GI**	Gastrointestinal
**HAC**	Hierarchical ascending classification
**IAA**	Indole-3-acetic acid
**LAB**	Lactic acid bacteria
**MATH**	Microbial adhesion to hydrocarbons
**MRS**	de Man, Rogosa and Sharpe
**NCBI**	National Center for Biotechnology Information
**PCA**	Principal component analysis
**PGPR/PGP**	Plant growth-promoting rhizobacteria/plant growth promotion
**SU**	Siderophore unit
**SD**	Standard deviation
