# Supplementary material for: Relevant Probiotic and Functional Properties of Lactic Acid Bacteria Isolated from Aquaculture Environments on the Ivory Coast for Potential Aquaponic Applications
Source: Microorganisms. 2026 Aug 28;14(9):1906. doi: 10.3390/microorganisms14091906 (PMC13609696; doi:10.3390/microorganisms14091906)
Supplement: Supplementary file 1 [file microorganisms-14-01906-s001.zip › microorganisms-4400039-supplementary.pdf]

Relevant Probiotic and Functional Properties of Lactic Acid Bacteria Isolated from Aquaculture Environments in Ivory Coast for Potential Aquaponic Applications

Wahauwouélé Hermann Coulibaly [1], Tano Marie-Ange Sakia Mian [2], Yabo Majoie Géroxie Tohoyessou [3], Muiz O. Akinyemi [4,5], Bassey Ebenso [4], Ange Olivier Parfait Yao [6], Cécile Meex [7], Paul-Alexandru Popescu [8], Thierry Fievez [9], Phillipe Maesen [9] and Hary Razafindralambo [6,10,11,12,*]

[1] Biotechnology and Food Microbiology Laboratory Food Science and Technology Formation and Research Unit, Nangui Abrogoua University, 02 PO Box 801 Abidjan 02, Côte d'Ivoire

[2] Agro-Valorization Laboratory, Agroforestery Trainning and Research Unit, Jean Lorougnon Guédé University, Daloa, BP-150, Ivory Coast, marieangemian@yahoo.fr

[3] Biology and Molecular Typage in Microbiology Laboratory, Biochemistry and Cell Biology Department Faculty of Sciences and Techniques, University of Abomey-Calavi, 05 PO Box 1604 Cotonou, Bénin

[4]Leeds Institute of Health Sciences, University of Leeds, Leeds, LS2 9LN, UK

[5]Unit for Environmental Sciences and Management, North-West University, Potchefstroom, South

[6]Microbial Processes and Interactions, TERRA Teaching and Research Centre, UMRt 1158 BioEcoAgro, Gembloux Agro-Bio Tech, University of Liege, Gembloux, Belgium

[7]University Hospital of Liege, Clinical microbiology laboratory, University of Liege, Belgium

[8]University of Agronomic Sciences and Veterinary Medicine of Bucharest,59 Marasti Blvd, District 1, Bucharest, Romania

[9]BEAGx, Gembloux Agro-Bio Tech, University of Liege, 2 Passage Des Déportés, B-5030, Gembloux, Belgium

[10]ProBioLab, 5004 Namur, Belgium

[11]An Giang University & Vietnam National University Hochiminh, Thu Duc, 71357, Ho Chi Minh city, Vietnam 18 Ung Van Khiem street, Long Xuyen, An Giang, 90100, Vietnam

[12]Université de Vakinankaratra, Ingénierie et Technologie Emergentes, Campus Vatofotsy Antsirabe 110 BP 108, Madagascar

*Corresponding author: h.razafindralambo@uliege.be

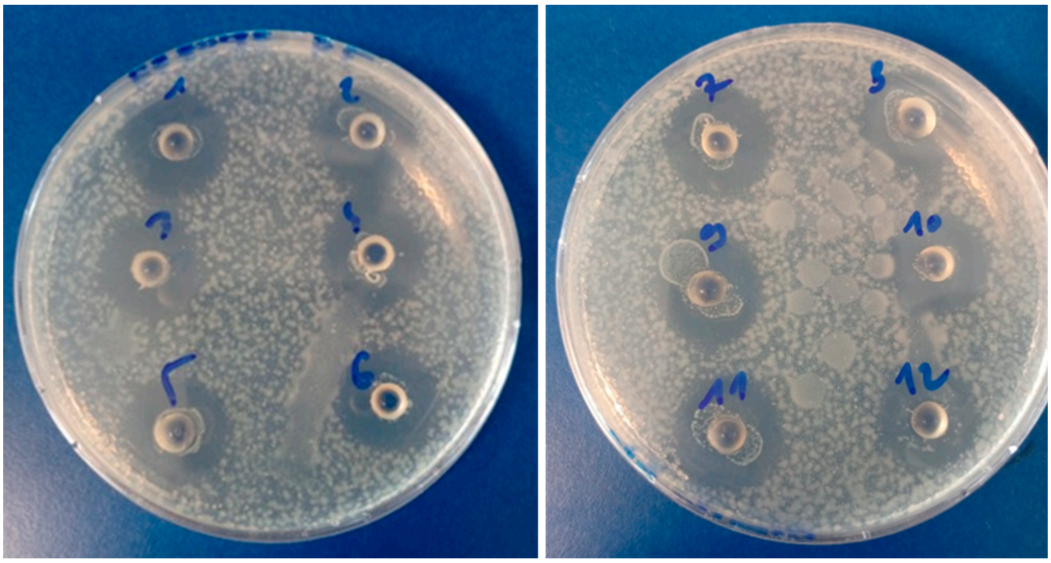

Figure S1. Illustration of the antibacterial activity agar plate of the 12 LAB isolates against *Pseudomonas aeruginosa* determined by well-diffusion assay. Inhibition is indicated by a clear zone.

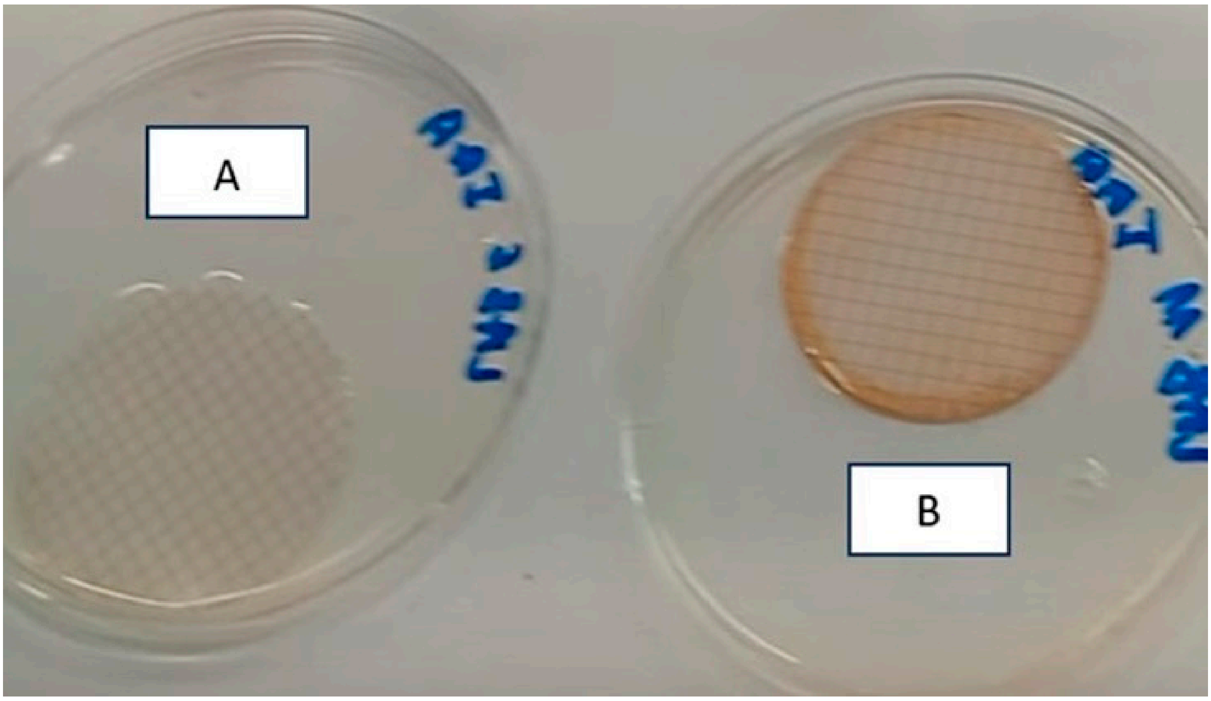

Figure S2. Indole-3-acetic acid (IAA) production was observed exclusively in LAB 11. A: negative reaction (no pink coloration). B: positive reaction (presence of pink colouration)

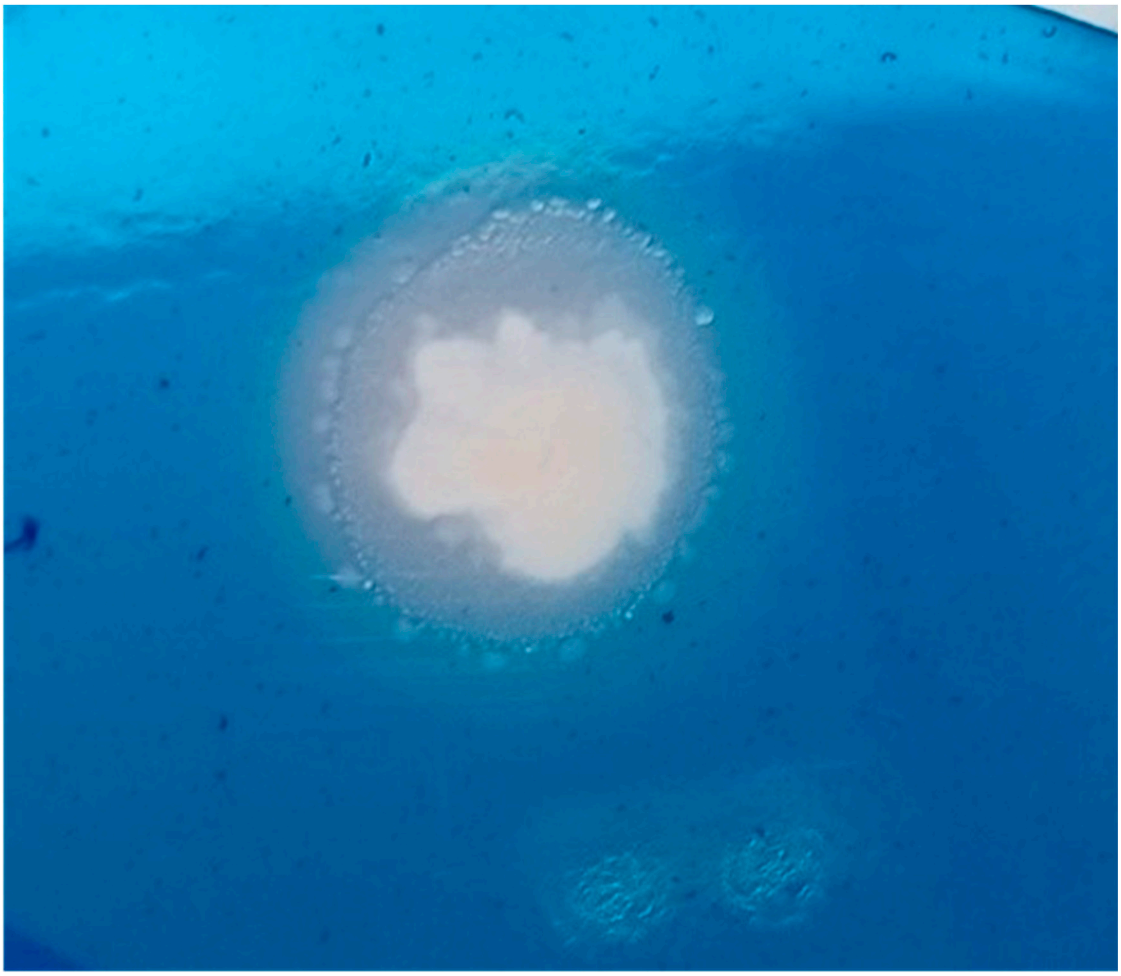

Figure S3: Representative agar plate showing siderophore production by LAB isolates

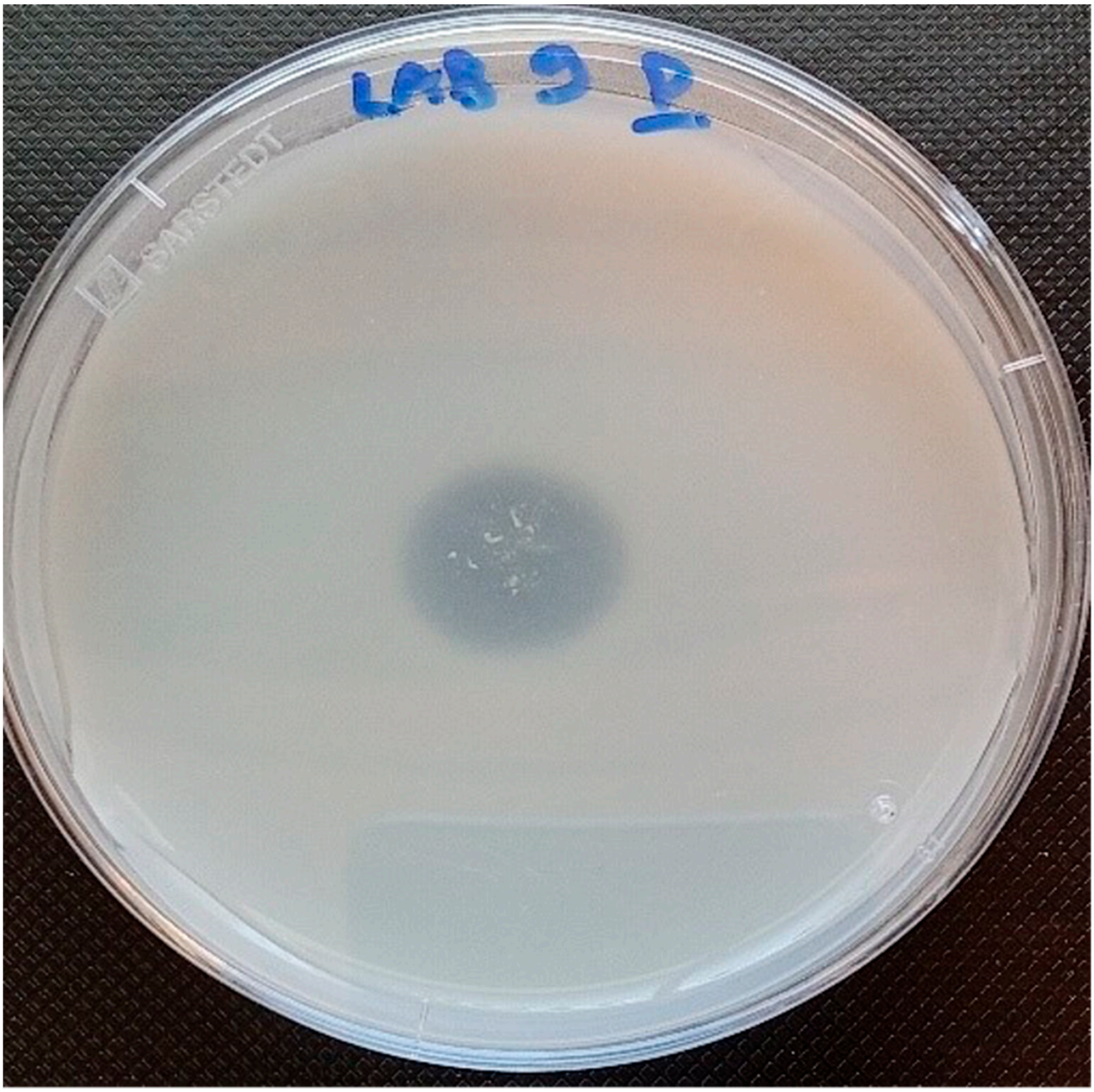

Figure S4: Representative Phosphate solubilisation activity of LAB strain

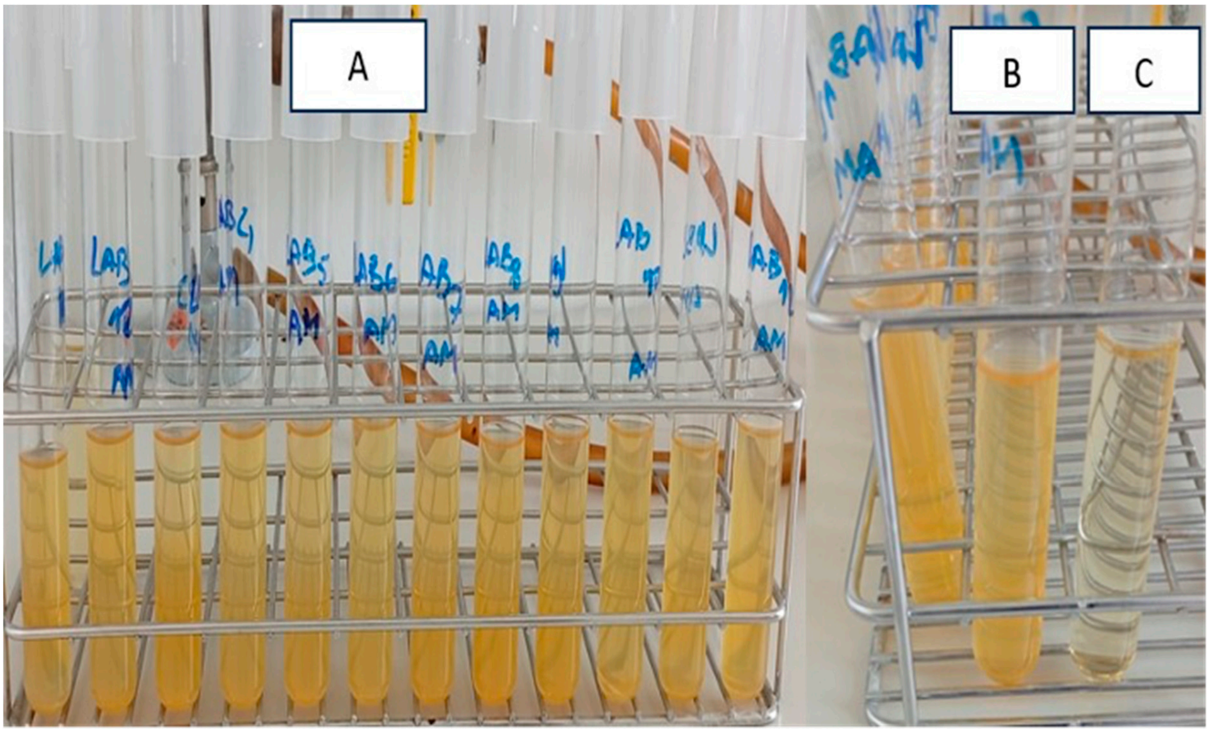

Figure S5. Representative ammonia production activity, A and B: positive reaction (yellow coloration); C: Negative control

Table S1: Antibiotic resistance profile and multiple antibiotic resistance (MAR) index across 12 LAB Strains. Resistance defined as non-susceptible (IR or R). Mean MAR index = 0.535, Median MAR index = 0.5

Strain	Number of Resistant Antibiotics	MAR Index
LAB 1	7	0.583
LAB 10	7	0.583
LAB 11	7	0.583
LAB 8	7	0.583
LAB 9	7	0.583
LAB 12	6	0.500
LAB 2	6	0.500
LAB 3	6	0.500
LAB 4	6	0.500
LAB 5	6	0.500
LAB 6	6	0.500
LAB 7	6	0.500

Table S2. Optical density, ammonia, nitrite, and nitrate concentrations from in vitro nitrification assay. Values are means of triplicate measurements (SD = 0 for ion concentrations, determined by kit calibration). Different lowercase superscripts indicate significant differences ($p < 0.05$, Tukey's test).

Strain	Ammonia (g/L)	Nitrite (mg/L)	Nitrate (mg/L)	OD_{600}
LAB 1	0.80	0.14	4	0.663 ± 0.005
LAB 2	0.80	<0.1	2	0.345 ± 0.021
LAB 3	0.75	0.10	2	0.636 ± 0.012
LAB 4	0.70	0.12	2	0.797 ± 0.007
LAB 5	0.80	<0.1	2	0.430 ± 0.003
LAB 6	0.90	<0.1	<2	0.491 ± 0.001
LAB 7	0.75	0.16	2	0.410 ± 0.002
LAB 8	0.70	<0.1	2	0.425 ± 0.014
LAB 9	0.75	<0.1	2	0.855 ± 0.003
LAB 10	0.70	0.35	8	0.439 ± 0.004
LAB 11	0.80	<0.1	2	0.472 ± 0.005
LAB 12	1.54	0.10	2	0.582 ± 0.007
